# Micro–nano integrated platforms for osteoarthritis therapy: From spatial manipulation to cellular reprogramming

**DOI:** 10.1016/j.mtbio.2026.102963

**Published:** 2026-02-23

**Authors:** Zhijian Shi, Jiayou Chen, Haochi Lun, Rongji Liang, Jingtao Huang, Quan Lin, Wei Li, Zhenhan Deng, Jianjing Lin

**Affiliations:** a Department of Sports Medicine and Rehabilitation, Peking University Shenzhen Hospital, PKU-Shenzhen Clinical Institute of Shantou University Medical College, Shenzhen, Guangdong, 518036, PR China; b Southern University of Science and Technology, Shenzhen, Guangdong, 518055, PR China; c Shantou University Medical College, Shantou, Guangdong, 515041, PR China; d Department of Orthopedics, The First Affiliated Hospital of Wenzhou Medical University, Nanbaixiang Road, Ouhai District, Wenzhou, Zhejiang, 325000, PR China; e Geriatrics Center, The First Affiliated Hospital of Wenzhou Medical University, Nanbaixiang Road, Ouhai District, Wenzhou, Zhejiang, 325000, PR China

**Keywords:** Osteoarthritis, Micro-nano composite structures, Drug delivery, Cartilage regeneration, Immunomodulation, Cell-free therapy

## Abstract

Clinical intervention for osteoarthritis (OA) has long been hampered by complex intra-articular physiological barriers, including rapid synovial clearance, the dense penetration resistance of the cartilage extracellular matrix (ECM), and a progressively deteriorating pro-inflammatory microenvironment. Conventional single-scale delivery systems frequently struggle to balance sustained retention with deep tissue penetration. Recently, micro-nano composite structures—engineered through the sophisticated integration of microscale matrices and nanoscale functional units—have catalyzed a paradigm shift from passive “space-filling” to active “fate modulation.” This review systematically delineates the recent advancements in micro-nano platforms for OA therapy. We first evaluate how advanced fabrication strategies, such as microfluidics, 3D bioprinting, and hierarchical emulsification, govern the spatiotemporal arrangement of these structures. Subsequently, we explore the mechanisms by which these trans-scale systems achieve prolonged joint residence, deep ECM infiltration, and precise immunomodulation of macrophages and stem cells. Furthermore, the roles of micro-nano architectures in recapitulating the biomimetic properties (topological, mechanical, and biochemical) of native cartilage and stimulating endogenous repair are highlighted. Finally, we provide a critical appraisal of the challenges hindering clinical translation, including scalability, biosafety margins, and the future of intelligent closed-loop designs. The development of micro-nano composite systems not only offers high-efficiency “cell-free therapy” for OA but also establishes a novel scientific paradigm for precision regenerative medicine in degenerative diseases.

## Graphical abstract

**Figure:**
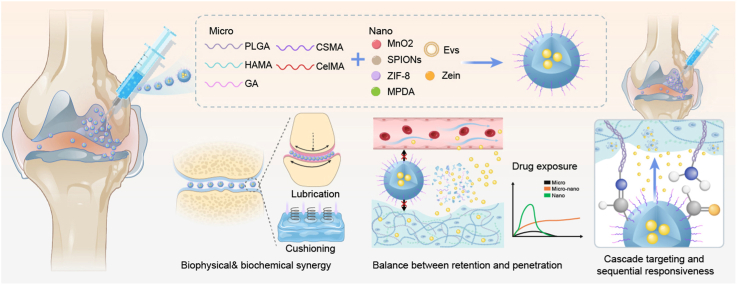

## 1 Introduction

Osteoarthritis (OA) is a highly complex, whole-joint degenerative disease characterized by a multifaceted pathological profile. This includes the progressive degradation of articular cartilage, aberrant subchondral bone remodeling, osteophyte formation, and chronic synovitis [1]. According to the Global Burden of Disease (GBD 2021) study, OA has emerged as a leading contributor to global years lived with disability (YLDs), with the affected population reaching 595 million in 2020 (approximately 7.6% of the global population) [2,3]. Driven by aging demographics and the rising prevalence of obesity (elevated BMI), the socio-economic burden of OA—including escalating healthcare expenditures and productivity loss—is increasing at an exponential rate [[2], [3], [4], [5]].

Current clinical interventions remain primarily palliative, focusing on symptomatic relief. Although oral non-steroidal anti-inflammatory drugs (NSAIDs) and intra-articular corticosteroid injections provide short-term pain management, they fail to reverse the pathological cascade of cartilage degeneration [6]. Notably, prolonged corticosteroid use may exacerbate cartilage attrition and secondary osteoporosis due to metabolic interference, while systemic NSAID administration is associated with significant cardiovascular, renal, and gastrointestinal toxicities [[7], [8], [9]]. While total joint arthroplasty remains the definitive intervention for end-stage OA, its clinical utility is constrained by the limited lifespan of prostheses and the inherent risks of revision surgery [3,10,11]. Consequently, there is an urgent clinical imperative to develop next-generation biomaterial platforms capable of delivering disease-modifying osteoarthritis drugs (DMOADs) with high precision and prolonged bio-retention [[12], [13], [14]].

Current OA therapies are fundamentally constrained by four distinct yet interconnected pathological barriers. First, the intrinsic reparative capacity of the tissue is severely limited. As a quintessential avascular, aneural, and alymphatic tissue, articular cartilage lacks the progenitor cell reservoir and nutrient supply required for self-repair following injury [15,16]. Mature chondrocytes constitute less than 5% of the tissue volume and are largely in a terminal differentiation state with low proliferative potential [17]. In the OA pathological milieu, the homeostatic balance of the extracellular matrix (ECM) is disrupted; the degradation rate of Type II collagen (COL II) and proteoglycans far exceeds their synthesis, culminating in the irreversible structural collapse of the cartilage framework [18,19]. Second, OA progression is driven by a complex, dynamic multicellular crosstalk that demands precise regulation [20]. The pathology extends beyond cartilage loss to involve the entire joint organ, characterized by a pro-inflammatory microenvironment dominated by M1 macrophage infiltration and the persistent secretion of cytokines (e.g., IL-1β, TNF-α), which upregulate matrix metalloproteinases (MMPs) and suppress pro-regenerative pathways [21,22]. This deterioration is further compounded by the accumulation of reactive oxygen species (ROS), which triggers mitochondrial dysfunction and accelerates chondrocyte senescence [17,[23], [24], [25]]. Consequently, effective therapy requires not merely a static scaffold, but a dynamic system capable of modulating these intricate intercellular interactions. Third, the uncoupling of biochemical signaling and biomechanical homeostasis creates a deleterious feedback loop. Crucially, the pathological coupling of aberrant joint stress distribution and excessive interfacial friction synergistically activates inflammatory signaling through mechanosensitive pathways (e.g., integrins and TRPV4), establishing a deleterious feedback loop where physical shear and mechanical strain exacerbate biochemical inhibition [26,27]. Conventional delivery systems frequently struggle to address this challenge by failing to synchronize mechanical support with biochemical modulation, leaving the regenerative tissue vulnerable to physical wear before biochemical cues can take effect [28]. Finally, while intra-articular injection offers a promising route for intervention, it faces a critical “Retention-Penetration” paradox. Therapeutic agents must overcome rapid synovial clearance to maintain effective concentrations [[29], [30], [31]]. Paradoxically, to reach the chondrocytes embedded within the dense, negatively charged ECM, agents must also possess deep penetration capabilities—a property often conflicting with the size and surface requirements for long-term retention. This dichotomy creates a significant bottleneck for single-scale drug delivery systems, necessitating the development of advanced platforms capable of simultaneously achieving macroscopic joint retention and microscopic tissue penetration [32].

To resolve these conflicting physiological constraints, Micro-Nano Composite Structures have emerged as hierarchical platforms that bypass the “Retention-Penetration” paradox through a strategic “Division of Labor.” By integrating micrometer-scale (1–100 μm) matrices with nanometer-scale (1–100 nm) functional units, the micro-framework serves as a “structural anchor” to withstand joint loading and resist synovial clearance, while embedded nano-units act as “penetration warheads” to infiltrate the dense ECM for precise cellular targeting [[33], [34], [35]]. This “nano-in-micro” biomimicry extends to microneedle (MN) patches, enabling painless transdermal delivery and localized drug deposition [[36], [37], [38]]. In the realm of cell-free therapy, these platforms bridge two distinct paradigms: bioactive factor delivery (e.g., exosomes, secretomes) and endogenous cell recruitment (in situ tissue engineering). While the former directly modulates the metabolic niche through the release of signaling molecules, the latter utilizes the scaffold's physicochemical properties to guide host progenitor cell homing and lineage direction. Ultimately, these systems catalyze a paradigm shift from passive “space-filling” to active “fate modulation.” Empowered by stimuli-responsiveness (e.g., pH/ROS) and magnetic targeting, they orchestrate spatiotemporal release to rebalance chondrocyte metabolism and direct macrophage polarization, facilitating the transition toward intelligent, closed-loop theranostics [[39], [40], [41]] (Fig. 3G and H).

**Fig. 1:**
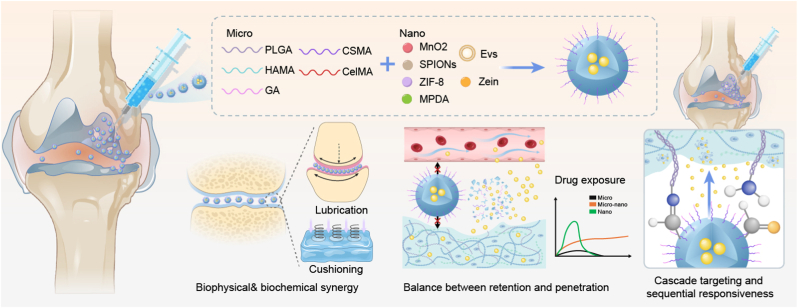
Conceptual framework of micro-nano composite structures for integrated osteoarthritis (OA) therapy. The hierarchical design integrates various micrometer-scale matrices (e.g., PLGA, HAMA, GA) with functional nanounits (e.g., MnO2, SPIONs, EVs) to achieve biophysical and biochemical synergy. Intra-articular injection of the composite system into the joint space. Biophysical functions, including boundary lubrication and mechanical cushioning, are integrated to re-establish joint homeostasis. The system achieves a "Retention-Penetration" balance, where the micro-framework ensures long-term joint retention while the released nano-units facilitate deep ECM penetration and intracellular drug exposure. Cascade targeting and sequential responsiveness, demonstrating how the hierarchical structure enables spatiotemporal drug delivery in response to the complex OA pathological microenvironment.

To provide a holistic view of these advancements, Fig. 1 illustrates the core design philosophy of micro-nano composite systems. By synergizing diverse micrometer-scale scaffolds (e.g., GelMA, HAMA) with functional nanometer-scale components, these platforms bridge the gap between biophysical support and biochemical modulation. This hierarchical architecture effectively resolves the “Retention-Penetration” paradox, ensuring that therapeutics not only persist within the joint space but also infiltrate the dense, avascular cartilage matrix for precise cellular uptake. Through sequential responsiveness and cascade targeting, these “nano-in-micro” systems offer a robust roadmap for transitioning from palliative care to disease-modifying, regenerative theranostics.

This review summarizes recent advances in fabricating these composite platforms and elucidates their therapeutic mechanisms, ranging from controlled drug delivery and lubrication to immunomodulation. Furthermore, we critically evaluate current translational hurdles, including scalability and biosafety, and discuss future directions for developing intelligent, closed-loop therapeutic systems.

## 2 Fabrication and structural design of micro-nano platforms

### **2.1** Micro-nano design: structural-functional synergy

**Fig. 2:**
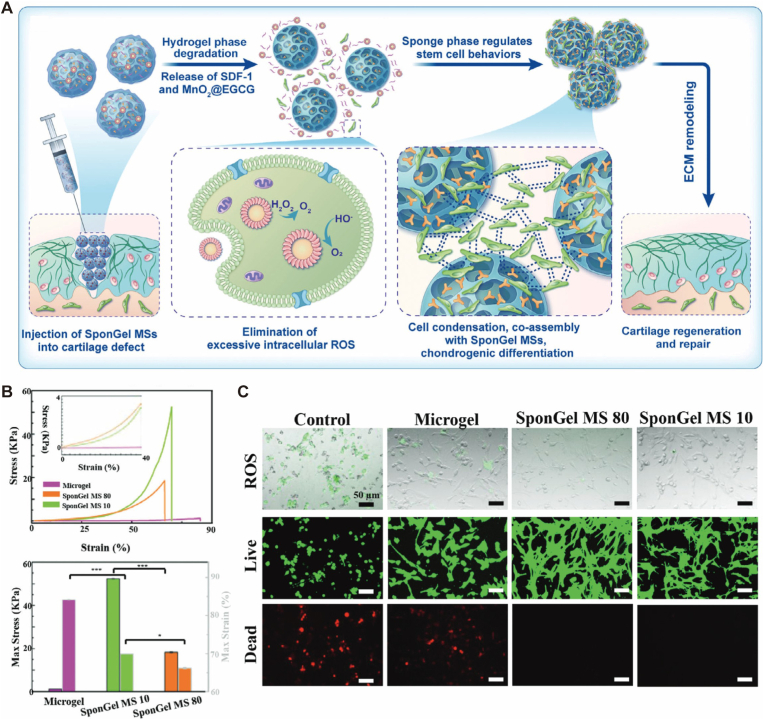
Hierarchical design of injectable SponGel microspheres for restoring cartilage homeostasis. (A) The biphasic strategy integrates a stable sponge framework for physical support with a dynamic hydrogel phase for nanoparticle delivery (MnO2@EGCG). (B) Mechanical evaluation demonstrates that the optimized pore structure (SponGel MS 10) significantly enhances compressive modulus to withstand physiological loading. (C) Biological performance showing that the functionalized microspheres effectively inhibit oxidative stress and support cell survival. This design exemplifies the synergy between micron-scale structural scaffolding and nanoscale bioactive regulation. Reproduced with permission[34]. Copyright 2025 John Wiley and Sons.

To circumvent the limitations of conventional intra-articular therapies—primarily rapid synovial clearance (residence <24 h), off-target toxicities, and the absence of structural support [7,31,42]—the development of micro-nano composite systems has evolved from simplistic drug carriers toward bio-integrated regenerative platforms. The design of these systems is governed by the following core principles:

Multiscale Hierarchical Mimicry and Mechanobiological Adaptation. By recapitulating the heterogeneous architecture of native articular cartilage, these systems create a biomimetic niche conducive to tissue repair. Micrometer-scale frameworks (20–100 μm pore size) emulate the spatial arrangement of collagen fibers, providing physical anchors for cells and optimizing nutrient diffusion [34,43]. Simultaneously, nanometer-scale units mimic the topological features of the extracellular matrix (ECM) to enhance intracellular delivery efficiency [[44], [45], [46]]. Such hierarchical designs, often incorporating biphasic structures, allow the mechanical properties (e.g., compressive modulus >50 kPa) to match those of native cartilage [34](Fig. 2B). Crucially, recent advances emphasize shifting from static stiffness matching to dynamic viscoelastic recapitulation. By engineering dynamic covalent linkages (e.g., Schiff base or hydrazine bonds), micro-matrices can be tuned to exhibit a stress relaxation time constant comparable to native tissue. This “stress-relaxing” capability enables the carrier to dissipate mechanical energy rather than transmitting destructive shockwaves to resident chondrocytes [28].

**Fig. 3:**
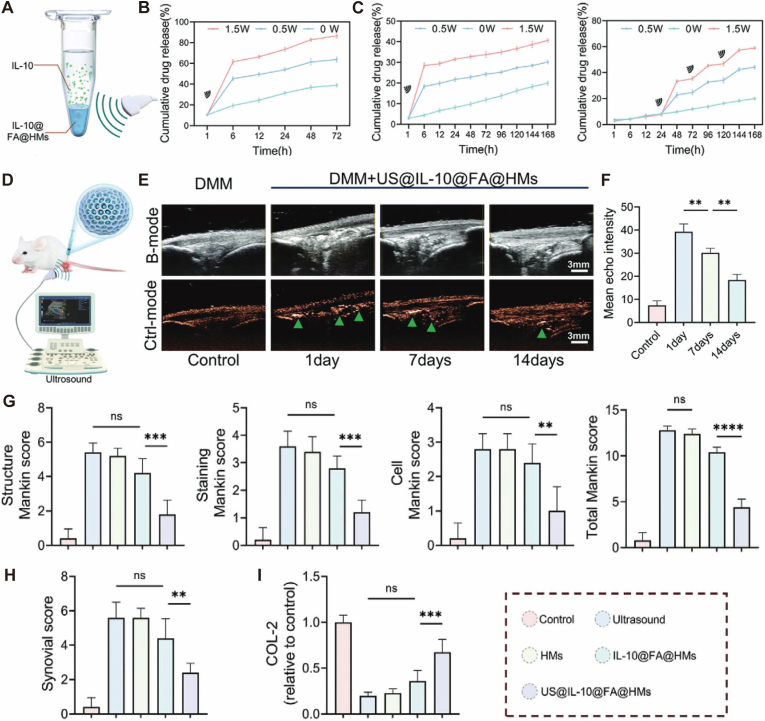
Construction and therapeutic evaluation of the ultrasound-responsive micro-nano composite system. (A-C) Stimuli-responsive release behavior of the micro-nano hybrids. The system comprises HAMA/GelMA microspheres (micro-scale scaffold) loaded with IL-10@PLGA nanobubbles (nano-scale functional units). Under ultrasound stimulation (1.5 W), the embedded nanobubbles undergo phase transition, triggering a burst release of IL-10 (reaching ∼40.67%) from the composite microspheres. (D-F) In vivo degradation monitoring. The micro-nano composite structure enables real-time ultrasound tracking. The gradual dissipation of the echo signal reflects the synchronized biodegradation of the micro-matrix, ensuring the carrier degrades compatibly with the tissue repair timeline. (G-I) Quantitative efficacy in joint repair. Treatment with the micro-nano composite system (US@IL-10@FA@HMs) significantly reduced Mankin (G) and Synovial (H) scores while enhancing COL-2 deposition (I). This demonstrates that integrating nano-scale precise delivery with micro-scale structural support effectively disrupts the inflammatory cycle and promotes hyaline cartilage regeneration. Reproduced with permission[47]. Copyright 2024 John Wiley and Sons.

However, the sophisticated design of these micro-nano platforms is intrinsically linked to their injectability, as robust shear-thinning properties are imperative for enabling minimally invasive delivery [48,49]. A critical design trade-off must be managed: the integration of stiff micro-scale matrices (e.g., PLGA microspheres) or high concentrations of inorganic nanounits to achieve the aforementioned mechanical robustness can inadvertently compromise rheological performance. For instance, a composite system containing 20% PLGA microspheres (∼30 μm) can exhibit an apparent viscosity 3-5 times higher than that of a base hydrogel, potentially necessitating larger-gauge needles (>20-gauge) that increase patient pain [[50], [51], [52]]. Consequently, balancing structural complexity with clinical injectability has become a foundational design criterion.

Spatiotemporally Controlled Degradation and Pathological Responsiveness. A critical design imperative is the synchronization of scaffold degradation with the kinetics of neo-tissue formation. By blending synthetic polymers (e.g., PLGA) with natural hydrogels (e.g., gelatin), these platforms achieve staged degradation profiles: rapid-release components address acute inflammation, while slowly-degrading matrices maintain long-term structural integrity for repair [42,47,53](Fig. 3D–F). Advanced iterations leverage endogenous triggers, such as MMP-13-sensitive peptides [54] or pH/ROS-responsive linkages [47,55,56], enabling “on-demand” drug release tailored to the inflammatory milieu, thereby maximizing therapeutic efficacy while minimizing off-target effects.

**Fig. 4:**
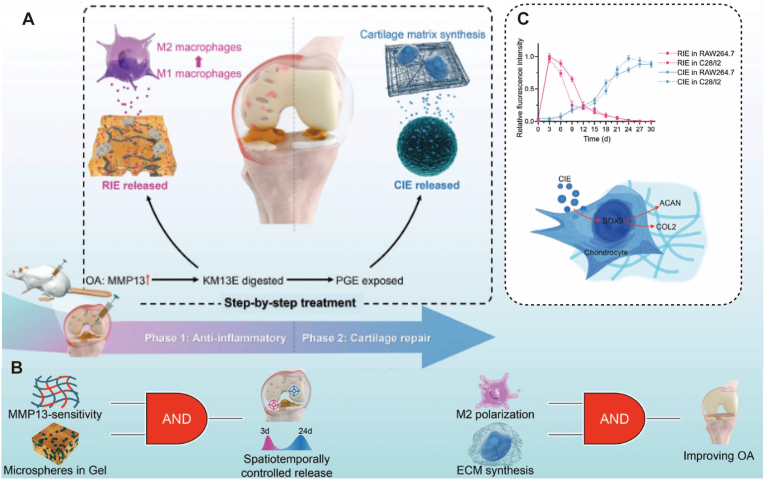
Sequential logic-gated “microspheres-in-gel” system for precise spatiotemporal coupling of anti-inflammation and cartilage regeneration. (A) Schematic illustration of the sequential therapeutic strategy responsive to the OA microenvironment. High levels of MMP13 (pathological trigger) degrade the outer self-assembled peptide hydrogel (KM13E) to rapidly release anti-inflammatory EVs (RIE) for macrophage M2 polarization (Phase 1: Anti-inflammatory). Subsequently, the inner gelatin methacryloyl microspheres (PGE) are exposed to sustainably release cartilage repair EVs (CIE) (Phase 2: Cartilage repair). (B) Schematic of the sequential logic (cascade) mechanism of the design principle. The system integrates enzyme sensitivity with a hierarchical structure to achieve a programmed "IF-THEN" response, ensuring that anti-inflammatory therapy (Phase 1) strictly precedes the cartilage repair phase (Phase 2). (C) Top: In vitro release profiles demonstrating the cascade-triggered kinetics: a burst release of RIE (pink) followed by a sustained release of CIE (blue) over 30 days. Bottom: The molecular mechanism wherein the delivered CIE promotes chondrocyte extracellular matrix synthesis (COL2 and ACAN) via the SOX9 pathway. This strategy exemplifies how biomaterial structural logic can be deeply coupled with pathological progression to maximize therapeutic efficacy. Reproduced under terms of the CC-BY license[54]. Copyright 2024, The Authors, published by Wiley‐VCH GmbH.

Functional Modularization and Translational Feasibility. Modern micro-nano platforms aim to integrate physical shielding, immune modulation, and lineage-specific differentiation into a unified system. Through modular construction, these carriers can sequentially reprogram the immune microenvironment and activate pro-chondrogenic pathways [19,24,57,58]. For instance, advanced “microspheres-in-gel” systems utilize sequential logic-gated designs to achieve precise spatiotemporal coupling. In this triggered-cascade architecture, pathological triggers (e.g., MMP13) initiate a rapid anti-inflammatory response (Phase 1), which subsequently exposes the internal matrix to facilitate sustained chondrogenic induction (Phase 2) (Fig. 4A、B). Furthermore, translational considerations have become central to the design process, focusing on high-fidelity manufacturing via microfluidics [59,60] and the incorporation of diagnostic elements, such as superparamagnetic nanoparticles for MRI tracking [61], moving towards a comprehensive theranostic approach for OA management.

### 2.2 Common materials for micro-nano composite structures

The superior therapeutic performance of micro-nano composite carriers stems from the precise architectural coupling of micrometer-scale matrices and nanometer-scale functional units. As the structural foundation of the system, the micrometer-scale matrix provides essential anchorage sites for cells and regulates the biochemical milieu through tailored degradation kinetics and mechanical robustness.

#### 2.2.1 Natural polymeric micrometer-scale matrices

Natural polymers are preferred candidates for constructing micrometer-scale scaffolds due to their exceptional biocompatibility, enzymatic degradability, and high structural resemblance to the native extracellular matrix.

1. Hyaluronic Acid (HA) and its Derivatives: As a hallmark component of the cartilage matrix, HA provides critical lubrication and reduces frictional coefficient [62,63]. However, unmodified HA hydrogels typically lack the requisite toughness and are susceptible to rapid degradation under high-shear intra-articular conditions. Methacrylated HA (HAMA) retains the intrinsic bioactivity of HA while introducing photo-crosslinking capabilities, which substantially enhances its structural stability under dynamic mechanical loading [39,64]. Microfluidic fabrication of monodisperse HAMA microspheres allows for precise control over internal porosity, thereby optimizing drug diffusion and endogenous cell infiltration [34,40,57]. Often serving as the “host” for composite structures, HAMA-based matrices integrated with POSS-DS nanoparticles or KGN-liposomes function as multifunctional scaffolds capable of simultaneous lubrication, controlled release, and chondrogenic induction [[64], [65], [66]].
2. Chitosan (CS) and its Derivatives: Chitosan is a natural cationic polysaccharide characterized by inherent anti-inflammatory activity and biodegradability. Methacrylated chitosan (CSMA) forms a dense, ECM-like nanofibrous network upon crosslinking, providing a 3D microenvironment that supports mesenchymal stem cell (MSC) adhesion and initiates chondrogenic signaling [33,44,65]. Regarding fabrication strategies, spray drying is frequently utilized for large-scale industrial production [67], whereas microfluidics enables the synthesis of uniform microspheres (100-500 μm) for the encapsulation of exosomes or hydrophobic nanodrugs, achieving synergy between immunomodulation and cartilage repair [34,57].
3. Dextran Sulfate and Chondroitin Sulfate: These glycosaminoglycan mimetics offer both structural support and targeted regulatory functions. Specifically, dextran sulfate microspheres are recognized by Dectin-1 receptors on macrophages, triggering receptor-mediated endocytosis for the targeted delivery of therapeutic agents (e.g., methotrexate or vanillin) [68,69]. Studies demonstrate that these microspheres effectively remodel the inflammatory niche by suppressing TNF-α and IL-6 levels, exhibiting anti-arthritic efficacy superior to traditional systemic administration [[70], [71], [72], [73], [74]].
4. Fibrin Microspheres: Plasma-derived fibrin possesses abundant cell-adhesion motifs (e.g., RGD sequences) and exhibits plasmin-responsive degradation [15]. The degradation kinetics of fibrin-based systems can be precisely tuned by adjusting fibrinogen concentrations or incorporating anti-fibrinolytic agents. When loaded with chondrogenic factors such as TGF-β3 or KGN, these microspheres significantly enhance MSC differentiation and ECM synthesis [23,53,75]. Furthermore, fibrin microspheres are frequently combined with chondroitin sulfate nanoparticles to emulate the hierarchical multi-component architecture of native cartilage, thereby facilitating in situ defect repair [15].

#### 2.2.2 Synthetic polymeric micrometer-scale matrices

Compared to their natural counterparts, synthetic polymers offer superior processability, mechanical stability, and precisely programmable degradation kinetics, rendering them indispensable for long-term drug delivery and biomechanically-tailored scaffolds [76].

1. Poly(lactic-co-glycolic acid) (PLGA): As an FDA-approved aliphatic polyester, the primary advantage of PLGA lies in its tunable degradation profile. By modulating the molar ratio of lactic acid (LA) to glycolic acid (GA), the degradation period can be precisely calibrated from weeks to several months [42,56]. For instance, GA-rich formulations (50:50) exhibit accelerated degradation, ideal for rapid drug release during acute inflammatory phases. Conversely, LA-rich variants (e.g., 75:25) provide enhanced hydrophobicity and structural longevity, better suited for the sustained regenerative requirements of cartilage repair. The degradation by-products, LA and GA, are cleared via the tricarboxylic acid (TCA) cycle, with clinical evidence confirming the minimal immunogenicity of PLGA following intra-articular injection [55]. In micro-nano engineering, the chemical robustness of PLGA facilitates high-efficiency encapsulation of small molecules (e.g., DXM), growth factors, or magnetic nanoparticles (e.g., SPIONs), enabling the construction of multifunctional systems with integrated therapeutic and imaging capabilities [42,44,56].
2. Gelatin Methacryloyl (GelMA) Microspheres: GelMA synergizes the biological motifs of natural gelatin (e.g., RGD sequences) with the tailorability of synthetic crosslinkable materials. Upon light-induced free-radical polymerization, GelMA rapidly forms a stable 3D hydrogel network [39,57,65]. In the context of OA therapy, GelMA microspheres are frequently employed as dynamic bio-active matrices for hierarchical subsystems to achieve complex sequential release logic. For example, embedding pH-sensitive liposomes or chemokines (e.g., SDF-1) within the GelMA matrix can trigger in situ recruitment and lineage-specific differentiation of endogenous stem cells by mimicking microenvironmental cues [34,57]. Furthermore, APPA-loaded GelMA microspheres have been shown to activate the Fgfr2 pathway, significantly enhancing chondrocyte proliferation and ameliorating joint damage in vivo [77].
3. Polycaprolactone (PCL): PCL is distinguished by its exceptional flexibility, low glass transition temperature, and exceptionally slow hydrolytic degradation rate (typically 1-2 years), making it a cornerstone for long-term delivery and load-bearing scaffolds [33,53]. Given its versatile processing properties, PCL is often utilized in electrospinning to mimic collagenous nanofibrous networks or in 3D printing to fabricate customized, porous platforms for cartilage repair [31,44]. In micro-nano composite architectures, microfluidic-engineered PCL microspheres exhibit minimal burst release of hydrophobic agents (e.g., DXM), serving as an ideal reservoir for the prolonged management of chronic inflammation in OA [33].

#### 2.2.3 Nanoscale functional units: inorganic nanoparticles

Inorganic nanoparticles, characterized by their unique magnetic responsiveness, catalytic activities, and environmental sensitivities, endow micro-nano composite systems with intelligent features for precision modulation and microenvironmental remodeling.

1. Superparamagnetic Iron Oxide Nanoparticles (SPIONs): SPIONs, primarily composed of Fe3O4 or γ-Fe2O3, are functional materials exhibiting high magnetic susceptibility without remanence. When incorporated into micrometer-scale matrices such as PLGA or HAMA, SPIONs facilitate the construction of magnetically-targeted delivery platforms [78,79]. A pivotal advantage of magnetic-responsive technology is its ability to overcome the rapid clearance effect of synovial fluid. Studies have demonstrated that magnetic field assistance extends the intra-articular residence time of SPION-loaded microspheres to 14 days, providing a stable reservoir for sustained anti-inflammatory therapy (e.g., DXM) [55,56]. Furthermore, SPION-modified systems support real-time MRI tracking and enable the magnetic guidance of MSCs to specific cartilage defects, significantly enhancing COL II deposition and structural restoration [80].
2. Manganese Dioxide (MnO2) Nanoparticles: As promising “nanozymes,” MnO2 nanoparticles exhibit exceptional efficacy in modulating the oxidative stress microenvironment of the OA joint. Porous MnO2architectures, synthesized via redox reactions, act as catalytic centers to decompose hydrogen peroxide and scavenge hydroxyl radicals, thereby arresting oxidative damage [24,34,81]. The underlying biological mechanism involves the reprogramming of macrophage polarization from the pro-inflammatory M1 phenotype to the pro-regenerative M2 phenotype through ROS scavenging [54,82]. For instance, hybrid systems incorporating MnO2@EGCGnanoparticles achieve a dual-function of “inflammatory control and stem cell recruitment,” creating an optimized, low-oxidative niche for in situ cartilage regeneration [10](Fig. 2A).
3. Hydroxyapatite (nHA) Nanoparticles: nHA mimics the inorganic phase of native bone tissue, offering superior bioactivity and osteo/chondro-inductivity. Depending on clinical requirements, nHA crystals can be synthesized into rod-like, plate-like, or spherical morphologies to optimize surface area and therapeutic efficacy [10,83]. Within composite delivery systems, nHA frequently serves as a controlled-release platform for hydrophobic molecules like Kartogenin (KGN). These nHA-KGN systems not only facilitate linear, long-term release kinetics but also safeguard stem cell bioactivity by emulating the native calcium-phosphate microenvironment [75].
4. Zeolitic Imidazolate Frameworks (ZIF-8) and Mesoporous Polydopamine (MPDA): Emerging responsive carriers have garnered significant interest for OA therapy. ZIF-8, characterized by its pH-sensitivity, rapidly dissociates in the acidic inflammatory milieu (pH < 6.5) to achieve targeted drug release [84]. In contrast, MPDA nanoparticles are ideal candidates for long-term reservoirs due to their exceptional drug-loading capacity (up to 87%) and the intrinsic anti-inflammatory and anti-apoptotic properties of the polydopamine backbone. Research indicates that MPDA-based systems can sustain therapeutic release for over 28 days, effectively preventing proteoglycan loss and maintaining cartilage homeostasis [10].

#### 2.2.4 Nanoscale functional units: organic and biopolymeric nanoparticles

Organic nanoparticles, characterized by their high degree of surface functionalization, excellent biodegradability, and superior encapsulation efficiency for hydrophobic agents, serve as the primary components for biochemical signaling modulation within micro-nano composite systems.

1. Zein Nanoparticles: Zein, a naturally occurring hydrophobic prolamine derived from maize, exhibits exceptional film-forming and self-assembly capacities [76]. In OA therapy, Zein nanoparticles offer a protective barrier for therapeutic cargo, effectively mitigating the risk of burst release for hydrophobic molecules [85]. Utilizing the polyelectrolyte-protein complexation method (leveraging electrostatic interactions with oppositely charged polysaccharides), uniform particles of approximately 150 nm can be fabricated [18]. Recent evidence underscores that the inherent hydrophobicity of Zein facilitates strong interactions with type II collagen, significantly extending intra-articular residency, while its pH-sensitive swelling enables site-specific drug release within the acidic inflammatory microenvironment [86,87]. Studies have demonstrated that chondroitin sulfate-loaded Zein NPs promote cartilaginous ECM synthesis while ensuring long-term safety, with degradation by-products (amino acids) actively suppressing pro-inflammatory cytokines like TNF-α [87]. Unlike many synthetic polymers, Zein provides an eco-friendly and cost-effective platform reducing production costs compared to PLGA for lipophilic drug delivery without requiring complex organic solvent systems [18,87].
2. Polymeric Polyester (PLGA/PCL) Nanounits: PLGA and PCL remain the archetypal synthetic polyesters for nanomedicine, playing indispensable roles in growth factor delivery and the construction of biomimetic scaffolds. PLGA nanoparticles, typically synthesized via double-emulsion or spray-drying techniques, often serve as the “functional core” of multi-component systems. For instance, chitosan-coupled PLGA-KGN systems significantly extend the intra-articular residence time of Kartogenin [44,55,56,75,88]. Simultaneously, PCL nanofibers fabricated via electrospinning recapitulate the nanotopographical cues of native collagen fibers. Experimental evidence confirms that such hybrid PCL/PLGA nanosystems exhibit superior potency in upregulating MSC differentiation markers, including COL II and aggrecan, compared to single-component polyester systems [33,58].
3. Phospholipid Nanoparticles (Liposomes): Phospholipid-based nanoparticles, including conventional liposomes and their functionalized derivatives, utilize their bilayer structure to provide a biomimetic encapsulation environment [89]. Beyond passive sustained release, surface modifications endow these vesicles with targeting specificity and environmental responsiveness. A prominent example is the WDLKG system, which integrates cationic DOTAP liposomes with cartilage-targeting peptides (e.g., WYRGRL) [57,90,91]. Expanding this to metabolic intervention, Zhao et al. (2024) engineered cartilage-targeted liposomes (DWRVIIPPRPSA-modified) that co-deliver siRNA and TGF-β to modulate cholesterol metabolism, achieving deep matrix penetration and effectively restoring 89% of damaged cartilage thickness via SREBP2/SOX9 signaling regulation [91]. When embedded within hydrogel microspheres, this system demonstrates exceptional tissue affinity and pH-responsive release within the inflammatory milieu, establishing a highly efficient molecular targeting platform for precision OA intervention [57,90].
4. Chitosan Nanoparticles: As the representative natural cationic polysaccharide, chitosan nanoparticles leverage their abundant amino groups for the high-efficiency loading of chondrogenic inducers via ionotropic gelation or covalent coupling. Their unique mucoadhesive properties facilitate prolonged interactions with the cartilage matrix [92]. By enhancing bioavailability and providing a bio-interactive surface for the composite microspheres, chitosan nanoparticles synergistically promote in situ regeneration of damaged articular cartilage [44,60].

#### 2.2.5 Nanocomposites: functional integration and precision modulation

Nanocomposites, engineered via sophisticated chemical synthesis and molecular assembly, integrate multiple functional modules within a single nanoscale entity [93]. This structural evolution marks a transition from passive “physical encapsulation” to active “chemical conjugation,” providing more precise toolkits for immunomodulation and in situ tissue repair in OA management.

1. Peptide Dendrimer-based Nanoparticles: Peptide dendrimers, characterized by their highly branched, symmetric architectures and dense surface functionalities, provide an ideal scaffold for high-density drug loading and site-specific modification. In a departure from conventional physical entrapment, researchers have employed covalent grafting to conjugate methotrexate (MTX) onto the dendrimer surface (G-MTX), followed by microfluidic encapsulation within hyaluronic acid (HA) microparticles [94,95]. This hierarchical arrangement not only endows the platform with responsiveness to the acidic inflammatory microenvironment but also significantly enhances the intracellular delivery efficiency of MTX. Consequently, it effectively drives the polarization of synovial macrophages from a pro-inflammatory M1 phenotype toward a pro-regenerative M2 phenotype, thereby remodeling the inflammatory milieu at its source [94,95].
2. Polydopamine-Magnesium Composite Nanoparticles (DIC/Mg-PDA NPs): The construction of DIC/Mg-PDA nanoparticles via metal-organic chelation exemplifies the paradigm of synergistic therapy. This system leverages the chelation between magnesium ions (Mg^2+^) and the catechol groups of polydopamine (PDA) to stabilize the non-steroidal anti-inflammatory drug (NSAID) diclofenac (DIC) [81,96]. Upon integration into injectable hydrogel microspheres, the sustained release of Mg^2+^collaborates with the intrinsic antioxidant properties of PDA to promote M2 macrophage polarization. More importantly, Mg^2+^ acts as a potent chondrogenic inducer, activating specific signaling pathways to direct the differentiation of bone marrow mesenchymal stem cells (BMSCs) into chondrocytes. This integrated approach achieves a dual biological effect of “inflammation suppression” and “structural restoration” at the molecular level [81,96].

#### 2.2.6 Natural nanostructures: extracellular vesicles and apoptotic bodies

Natural nanostructures, particularly cell-derived lipid bilayer vesicles, have emerged as cornerstones of cell-free therapy for OA, attributed to their low immunogenicity, exceptional biocompatibility, and intrinsic multi-target regulatory capacity. Unlike synthetic counterparts, these biological entities harbor a complex repertoire of endogenous bioactive molecules, enabling precise, multi-pathway tissue restoration without the need for exogenous drug loading.

1. Mesenchymal Stem Cell-derived Extracellular Vesicles (MSC-EVs): MSC-EVs, including exosomes and microvesicles, circumvent the risks of immune rejection and embolic complications associated with direct cell transplantation while delivering lineage-specific biological cues [65,[97], [98], [99]]. EVs from diverse cellular origins exhibit distinct reparative profiles. For instance, adipose-derived EVs (ADSC-EVs) are enriched with specific microRNAs (e.g., miR-486-5p) that suppress chondrocyte apoptosis and drive M2 macrophage polarization [58,100]. Synovial or bone marrow-derived EVs enriched with superoxide dismutase 3 (SOD3) effectively maintain ECM homeostasis by scavenging ROS and downregulating matrix-degrading enzymes (MMP13 and ADAMTS5) [24,97]. Furthermore, peripheral blood-derived “hypACT” EVs significantly upregulate the synthesis of COL II and aggrecan [97,100]. To address challenges in yield and kinetic control, microfluidic platforms have been employed to enhance EV production while preserving biological activity via low-shear processing [24,97]. Recent innovations, such as the “logic-gated” KM13E@PGE system, utilize MMP-13 responsiveness to achieve sequential release of anti-inflammatory and pro-regenerative EVs, thereby synchronizing therapeutic intervention with the evolving pathological stages of OA [54].
2. Engineered Apoptotic Bodies (eABs): As a burgeoning class of natural delivery platforms, eABs offer unique advantages in terms of scalable production and innate chemotactic recognition. T cell-derived ABs, for example, can be expanded exponentially in vitro while retaining the inherent tropism of their parent cells toward inflammatory foci [101]. Fabrication and Functionalization: Triggered by UV irradiation or chemical induction, eABs can be further modified via membrane fusion or covalent coupling. When integrated into hydrogel microspheres, these platforms ensure prolonged localized retention and sustained bio-cargo release. Biological Predominance: In contrast to exosomes, ABs are characterized by the abundant surface expression of “eat-me” signals, such as phosphatidylserine (PS) [102]. This molecular signature facilitates their preferential uptake by macrophages via efferocytosis, leading to highly efficient immunomodulation [101]. Such composite systems provide both mechanical lubrication and a bio-active niche conducive to in situ cartilage repair.

The biomaterials employed in these platforms are categorized based on their hierarchical scales and origins, ranging from natural/polymeric microspheres that provide structural residence to inorganic/bio-derived nanounits for precise therapeutic intervention, as detailed in Table 1.

**Table 1:** Classification and functional characteristics of various biomaterials in micro-nano composite platforms for OA therapy.

Scale	Category	Representative Materials	Key Functional Roles	Core Physicochemical Advantages	Ref.
**Micro-sphere**	**Natural Polymers**	**GelMA**	Micro-scaffold/Cell carrier	Photo-crosslinkable; mimics natural ECM	[39,53]
		**Hyaluronic Acid**	Lubrication/Targeted delivery	CD44-mediated targeting; friction reduction	[47,103]
		**Chitosan**	Encapsulation/Surface coating	Cationic nature; enhanced mucoadhesion	[29,81]
	**Synthetic Polymers**	**PLGA**	Controlled release core	FDA-approved; precisely tunable degradation	[57,59]
		**PCL**	Mechanical reinforcement	High structural integrity; long-term stability	[43,104]
		**PEG**	Hydrophilic modification	Stealth effect; reduced non-specific adsorption	[55,60]
**Nano-unit**	**Inorganic Materials**	**Nano-HAp**	Interface biomimicry	Promotes mineralization; enhances modulus	[24,75]
		**MnO2**	Oxygenation/Anti-inflammation	ROS scavenging; in situ oxygen generation	[105]
		**Nanoclay**	Rheological modifier	Shear-thinning; improves bioprintability	[33,34]
	**Bio-derived Materials**	**Exosomes**	Bioactive cargo	Endogenous miRNA delivery; low immunogenicity	[54,58]
		**dECM**	Multi-signal reservoir	Preserves complex growth factors and cues	[18,104]
	**Responsive Units**	**SPIONs**	Magnetic targeting/Imaging	Field-guided accumulation; MRI tracking	[55,61]
		**Smart Hydrogels**	Logic-gated release	pH/Enzyme/Redox sensitive triggered delivery	[57,106]

### 2.3 Precision fabrication of integrated micro-nano systems

The therapeutic efficacy of micro-nano composite carriers depends not only on material selection but also on the precision of fabrication strategies in governing the spatial arrangement and functional coupling of micrometer-scale matrices and nanometer-scale units. Through programmed design of fluid dynamics, phase transition kinetics, and printed topologies, these strategies enable the realization of monodisperse morphologies, linearized release kinetics, and biomimetic mechanical properties.

#### 2.3.1 Microfluidics: programmed assembly of monodisperse microspheres

Leveraging its exceptional fluid manipulation capacity at the micrometer scale, microfluidics has emerged as the definitive technology for synthesizing highly monodisperse microspheres (100–500 μm in diameter). Utilizing droplet-based microfluidics, researchers can achieve the programmed distribution of nanoparticles within a micrometer-scale matrix via the droplet-template effect. For instance, by precisely adjusting the flow rate ratios of the aqueous/oil phases and channel geometries, the coefficient of variation (CV) for GelMA or HAMA microspheres can be maintained below 5%, significantly outperforming conventional bulk emulsification [57]. In multiscale integration, microfluidic platforms induce the homogeneous loading of nanounits (e.g., SPIONs or ZIF-8) during droplet solidification. This high-fidelity integration ensures that the encapsulation efficiency of chondrogenic inducers, such as Kartogenin (KGN), exceeds 90%, thereby providing an engineering foundation for standardized therapeutic parameters [57,60].

#### 2.3.2 Emulsification and spray drying: scalable synthesis of multiphase systems

Emulsification techniques (single/double emulsion) remain the classical approach for constructing polyester-based (PLGA, PCL) micro-nano carriers. Specifically, the double emulsion method (W/O/W) allows for the encapsulation of nanoscale liposomes within a micrometer-scale polyester shell, creating a “nano-in-micro” (NiMs) hierarchical release system. This architecture enhances local retention and reduces the initial burst release rate from 41% to 9%, effectively extending the therapeutic window [42,59]. Meanwhile, spray drying exhibits immense potential for the industrial translation of natural polymer-based carriers. Chitosan/KGN nanocrystal microspheres fabricated via this technique achieve immediate reconstitution and maintain stability within the intra-articular space for up to 14 days [60]. Furthermore, for thermolabile growth factors like TGF-β3, low-temperature spray drying combined with trehalose stabilizers limits bioactivity loss to below 15%, ensuring the functional integrity of bioactive molecules during processing [19].

#### 2.3.3 Electrospinning,3D bioprinting and microneedle fabrication: customized biomimetic architectures

To recapitulate the complex hierarchical architecture of native cartilage, electrospinning, 3D bioprinting and microneedle (MN) fabrication are widely utilized for developing multiscale biomimetic platforms [107]. Electrospinning generates ultrafine fibers (200–800 nm in diameter) by modulating electric field intensity and solution viscosity, creating ECM-like meshes to facilitate cell adhesion and matrix secretion [42]. Hybrid “sphere-in-fiber” scaffolds, formed by embedding PLGA-TGF-β3 microspheres within PCL fibrous networks, significantly upregulate the expression of chondrogenic markers [19]. 3D bioprinting further enables spatial customization from geometric macrostructures to functional micro-components. Utilizing high-precision extrusion, researchers can achieve the compartmentalized distribution of anti-inflammatory exosomes and chondrogenic microspheres within a single scaffold, thereby synchronizing immunomodulation and cartilage regeneration in both spatial and temporal dimensions [65].

Complementary to these scaffolds, MN fabrication has emerged as a specialized strategy for “nano-in-micro” integration. The synthesis of MN arrays typically employs template-assisted molding or centrifugal casting to precisely localize nanounits—such as drug nanocrystals [36], microemulsions [38], or exosomes [108]—within the tips of micro-scale polymeric needles (typically 200–800 μm in height). This structural compartmentalization ensures high loading efficiency and mechanical robustness, allowing therapeutic nano-cargoes to bypass physiological barriers and be released upon the programmed dissolution of the micro-needle matrix (e.g., HA or PVP) in the interstitial environment [37,109].

#### 2.3.4 Synergistic optimization of fabrication and function

In summary, the core of advanced fabrication integration lies in fulfilling the multifaceted clinical requirements of OA therapy through multiscale spatial coupling and spatiotemporally controlled distribution. This synergy is manifested in three primary dimensions.

First, the refinement of delivery kinetics: microfluidic platforms, “nano-in-micro” (NiMs) architectures and MN patches utilize hierarchical encapsulation to retard rapid drug clearance, while the intricate topologies enabled by 3D bioprinting allow for the programmable modulation of therapeutic release profiles [29,59]. Specifically, the precise structural control in MNs allows for the transition from traditional systemic administration to localized, painless transdermal delivery, significantly improving bioavailability [36,37].

Second, biomechanical adaptation and niche recapitulation: the biomimetic modulus (0.1–1 MPa) afforded by electrospinning, coupled with the gradient designs of 3D printing, effectively addresses the long-standing challenge of mechanical mismatch at the osteochondral interface [19,104]. Furthermore, the incorporation of responsive moieties or lubricating coatings into MN arrays demonstrates the feasibility of bridging mechanical lubrication with biochemical intervention [110].

Finally, the enhancement of translational feasibility: the scalability of spray drying combined with the mild processing conditions of microfluidics and the high patient compliance of MN patches provide a robust technological framework for the development of standardized, high-performance micro-nano composite systems [29,60]. Such a holistic optimization—bridging engineering precision with biological imperatives—represents the cornerstone for the next generation of precision OA intervention platforms.

The structural complexity and functional diversity of these platforms are realized through advanced fabrication strategies. A comparative analysis of these techniques, including microfluidics, 3D bioprinting, hierarchical emulsification, and micromolding-based microneedle (MN) fabrication is presented in Table 2. Each strategy offers unique advantages in terms of structural precision, scalability, and the ability to recapitulate the hierarchical architecture of native cartilage. Specifically, while microfluidics and 3D printing excel in spatial patterning within the joint space, MN fabrication provides a specialized “nano-in-micro” toolkit for bypassing tissue barriers through precision-molded needle arrays [36,37]. By integrating these diverse manufacturing approaches, researchers can bridge the gap between engineering precision and biological imperatives, providing a versatile toolkit for developing precision OA therapies.

**Table 2:** Comparison of advanced fabrication strategies and structural designs for osteoarthritis regenerative medicine.

Fabrication Strategy	Specific Techniques	Structural Characteristics	Key Advantages for OA Therapy	Ref.
**Microfluidics**	**Droplet Microfluidics**	Highly monodisperse NiMs	Precision size control; high encapsulation efficiency	[29,47]
	**Capillary Assembly**	Multicompartment spheres	Controlled spatial distribution of multiple drugs	[47,61]
**3D-Bioprinting**	**3D Extrusion Printing**	Macro-lattice scaffolds	Superior mechanical support; customized geometry	[19,104]
**Spinning**	**Electrospinning**	Nanofibrous membranes	Mimics ECM topology; high surface-to-volume ratio	[18,44]
**Microneedles**	**Micromolding & Centrifugal Casting**	Hierarchical arrays; mechanical robustness; customizable height.	Painless penetration; high bioavailability; improved compliance	[36,37,108]
	**Surface Coating**	Bio-inspired coatings	Lubricating-delivery dual functions; reduced insertion trauma	[38,110,111]
**Techniques**	**Solution Blowing**	Randomly oriented fibers	Rapid production; simplified operational process	[44,81]
**Emulsification**	**Double Emulsion**	Core-shell microspheres	Sequential release of hydrophilic/phobic drugs	[55,59]
	**Pickering Emulsion**	Solid-stabilized particles	High stability; reduced surfactant toxicity	[55,57]
**Drying & Deposition**	**Spray Drying**	Nanoclusters/Powders	Scalable industrial production; high throughput	[29,60]
	**Nanospray Drying**	Ultra-fine particles	Precise control over nanoparticle morphology	[60,101]

## 3 Precision OA therapy: trans-scale design and microenvironment repair

The effective treatment of osteoarthritis is hindered by complex physiological barriers within the joint, including rapid synovial clearance, the high penetration resistance of the dense cartilage extracellular matrix, and a progressively deteriorating pro-inflammatory milieu. Micro-nano composite systems leverage a trans-scale synergistic strategy—where micrometer-scale matrices provide macroscopic retention and nanometer-scale units achieve deep tissue penetration—alongside precision microenvironment remodeling via spatiotemporal responsive drug release and targeted immunomodulation. This approach establishes a therapeutic closed-loop from “reservoir retention” to “targeted responsiveness,” offering innovative solutions to the translational challenges of OA therapy [10,39].

### 3.1 Hierarchical platforms: long-term residency and deep infiltration

#### 3.1.1 Spatial compartmentalization and cascaded delivery

**Fig. 5:**
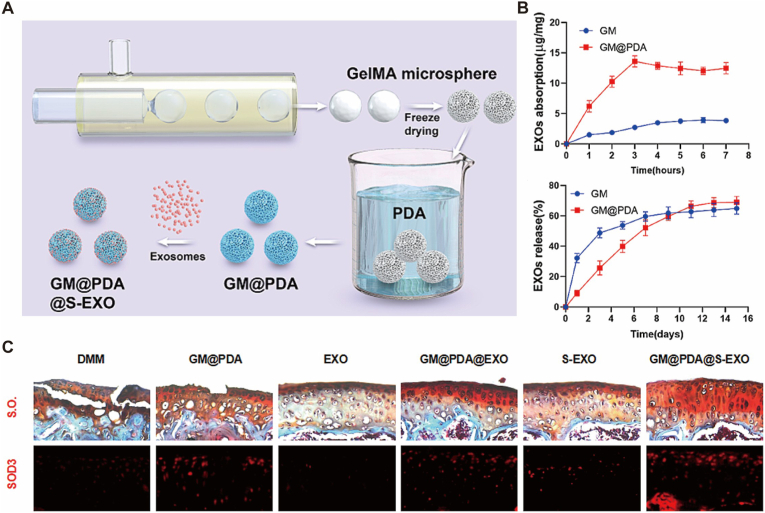
“Micro-reservoir - Nano-penetration” biphasic delivery system for deep cartilage permeation and OA therapy. (A) Schematic illustration of the hierarchical construction. Gelatin methacryloyl (GelMA) microspheres function as the “micro-reservoir” (surface-modified with polydopamine, PDA) to densely load and protect the “nano-penetrator” units—SOD3-enriched exosomes (S-EXOs). This design leverages the size effect of exosomes (30–150 nm) to penetrate the dense extracellular matrix (ECM). (B) Drug release kinetics showing the reservoir function. The GM@PDA system achieves high exosome loading capacity and provides a sustained release profile over 14 days, preventing rapid clearance from the joint cavity. (C) In vivo evaluation of cartilage penetration and protection. Top row: Safranin O staining (S.O.) indicates superior preservation of cartilage matrix and glycosaminoglycans in the composite system group (GM@PDA@S-EXO) compared to free exosomes. Bottom row: Immunofluorescence of SOD3 (red) reveals that the biphasic system successfully delivers antioxidant enzymes deep into the cartilage layers, overcoming the superficial retention limitation of free drugs. Collectively, this system utilizes the “micro-reservoir” for retention and “nano-penetration” for deep tissue access, significantly enhancing anti-inflammatory and antioxidant efficiency. Reproduced under terms of the CC-BY license[24]. Copyright 2024, The Authors, published by Springer Nature.

The superiority of micro-nano systems resides in their sophisticated spatial compartmentalization. Primary micrometer-scale matrices (e.g., HAMA or GelMA microspheres, 10–100 μm) effectively resist mechanical flushing by the synovial fluid through an increased hydrodynamic radius, extending intra-articular residence time to over 14 days [39,47]. This “micro-reservoir” function is validated by drug release kinetics, which demonstrate high loading capacity and a steady, sustained release profile that prevents the rapid clearance typical of simpler delivery vehicles (Fig. 5B). Furthermore, the negative surface charge of alginate microspheres induces electrostatic repulsion, significantly reducing phagocytic clearance by macrophages [42].

At the secondary responsive level, encapsulated nano units (50–200 nm liposomes or exosomes) utilize size-effect-driven transport to penetrate the dense ECM meshwork. Recent evidence confirms that SOD3-loaded exosomes (S-EXOs) can penetrate deep into the middle zone of cartilage, whereas free drugs remain sequestered at the surface [31,112] (Fig. 5C). This “micrometer reservoir-nanometer penetration” biphasic delivery increases drug penetration depth by 3-fold and enhances IL-1β inhibition efficiency by 164% compared to free drug controls [24,57] (Fig. 5B).

#### 3.1.2 Multi-target precision localization

Through precise surface functionalization, micro-nano architectures can achieve spatiotemporal targeting of OA pathological sites. Specifically, ligand-receptor interactions are frequently exploited to enhance cellular affinity; for instance, conjugation with the WYRGRL peptide has been shown to increase chondrocyte uptake by 8-fold [57]. Refining this approach for stage-specific intervention, Fei et al. identified that the Epidermal Growth Factor Receptor (EGFR) is exclusively overexpressed in early degenerating cartilage. By modifying boron nitride nanosheets with an EGFR-targeting peptide (CAP), they achieved selective therapeutic accumulation in OA lesions significantly higher than in healthy tissue, enabling precise early-stage treatment [113]. In parallel, to address the inflammatory microenvironment, folic acid (FA)-modified nanocarriers can specifically recognize M1 macrophages (increasing uptake by 4.2-fold) [47], while functionalization with CD44 antibodies facilitates the precise anchoring of synovial fibroblasts [112]. Building on these individual strategies, synergistic multi-target systems (e.g., FA/WYRGRL co-modification) have been developed to simultaneously drive macrophage phenotypic reprogramming (M1-to-M2) and upregulate matrix synthesis (3.1-fold increase in COL II), thereby achieving bidirectional “anti-inflammatory and regenerative” modulation [40,57] (Fig. 3I).

Beyond surface chemical conjugation, recent advances have leveraged genetically engineered cell membrane coatings to achieve “lock-and-key” precision targeting against specific pathological cell states. Wang et al. pioneered a dual-targeting strategy by coating nanoparticles with chondrocyte membranes genetically engineered to overexpress NKG2D receptors. These biomimetic nanounits can specifically recognize and anchor to MICA/B ligands—surface markers exclusively upregulated on senescent chondrocytes—thereby enabling the precise delivery of senolytic agents (ABT263) to clear “zombie cells” without affecting healthy tissue [114,115].

#### 3.1.3 Logic-gated and sequential cascaded release

To address the dynamic pathological features of OA, micro-nano systems are endowed with logic-gated responsiveness. Leveraging the acidic pH (<6.5) or elevated ROS levels in inflammatory zones, “self-feedback” carriers incorporating hydrazone bonds or MnO_2_ nanozymes achieve on-demand drug triggering [34,57]. For instance, MMP-13-sensitive hydrogels (e.g., KM13E) exhibit degradation rates 6 times higher in diseased tissue than in healthy controls [54]. Hierarchical structural designs (e.g., pH-responsive liposome-anchored hydrogel microspheres for KGN delivery) enable physiologically synchronized sequential release—rapidly counteracting the acidic inflammatory microenvironment followed by sustained chondrogenic induction. Animal models have demonstrated that this integrated scaffold strategy significantly reduces OARSI scores [57].

### 3.2 Biomechanical restoration: biomimetic lubrication and interfacial modulation

**Fig. 6:**
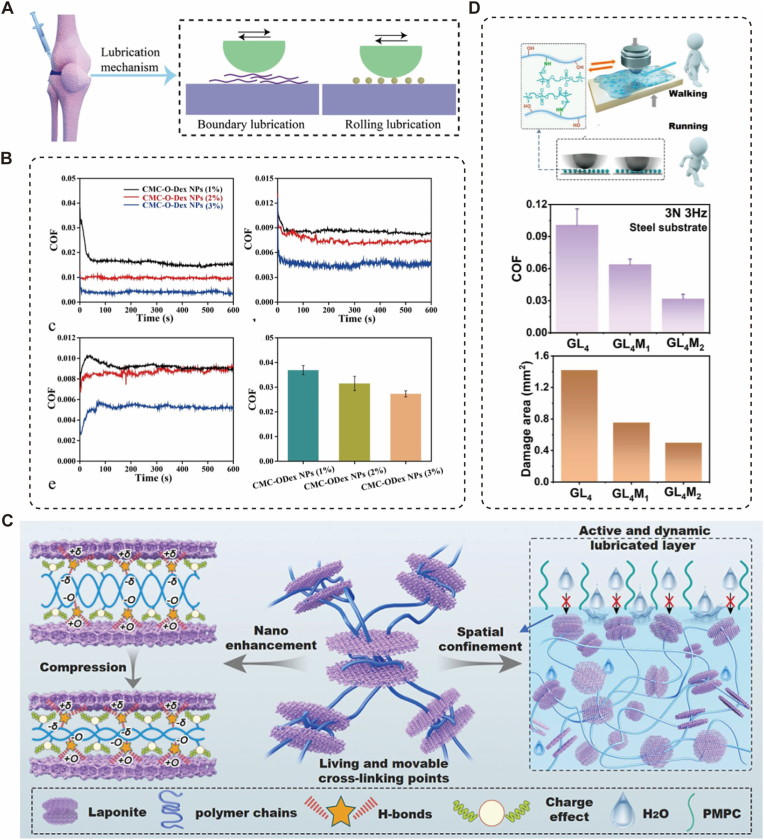
Biomimetic lubrication strategies based on micro/nano-composite architectures. (A) Schematic illustration of the injectable hydrogel network constructed by carboxymethyl chitosan and polymer-grafted nanospheres (TPN). The “hairy” nanospheres act as dynamic crosslinking nodes to dissipate energy. (B) The lubrication mechanism where the nanospheres function as hydration reservoirs and “ball bearings” to significantly reduce the coefficient of friction (COF) under sliding motion. Reproduced with permission[39]. Copyright 2023 Elsevier B.V. (C) Design of nano-confined hydrogel microspheres with programmable mechanics. The structure mimics the lubricin-hyaluronic acid complex on the cartilage surface. (D) Tribological performance analysis demonstrating that the surface hydration layers of the microspheres provide exceptional molecular lubricity, effectively decoupling stiffness from friction reduction. These designs collectively demonstrate how manipulating micro-nanostructures can effectively re-establish the low-friction physiological environment of articular joints. Reproduced with permission[116]. Copyright 2025 John Wiley and Sons.

Micro-nano composite structures function not only as biochemical signal carriers but also as “physical enhancers” of joint performance. In the reconstruction of boundary lubrication, advanced nano-confined hydrogel microspheres with programmable mechanics act as adaptive hydrostatic support units [116] (Fig. 6C). By generating interstitial fluid pressure within the composite network, these micro-nano structures provide essential biomechanical buffering that complements the native cartilage matrix [39]. Through the precise regulation of their internal nano-confinement effects, these microspheres achieve superior molecular lubricity and structural resilience under varying physiological loads [116] (Fig. 6D). This biomechanical synergy is further augmented by “hairy” nanosphere-grafted networks such as those employing polymer-grafted nanospheres (TPN) which act as dynamic crosslinking nodes to effectively dissipate energy during joint movement (Fig. 6A). Simultaneously, these units function as molecular “ball-bearings” that re-establish a low-friction surface hydration layer [39,116] (Fig. 6B), ensuring consistent joint mobility. Concurrently, nanolubricants released from these microspheres emulate native lubricin, forming a stable boundary film on the cartilage surface and reducing the friction coefficient to 0.02 [39,41]. By interrupting the mechanobiological NF-κB inflammatory pathway, this biomimetic system downregulates MMP-13 expression by 42.3%, effectively delaying cartilage degeneration from a biophysical perspective [24,106].

Crucially, the concept of “Regenerative Lubrication” has emerged to bridge the gap between passive friction reduction and active tissue repair. Han et al. engineered microfluidic microspheres functionalized with Nanofat (NF). These hierarchical units function as “rolling bearings” to achieve super-lubrication, yet uniquely, the NF coating acts as a bioactive depot. This design ensures that while the joint is mechanically protected from wear, the sustained release of adipose-derived growth factors actively modulates the inflammatory microenvironment, realizing the true synchronization of tribology and biology [117,118].

Complementing surface lubrication, the internal “shock absorption” capacity of the micro-matrix is pivotal for dampening deep-tissue mechanotransduction. Zhao et al. proposed a “Cellular Shock Absorber” strategy by constructing viscoelastic HAMA microspheres via dynamic hydrazine-aldehyde coupling. Unlike elastic scaffolds that store and recoil energy, these stress-relaxing matrices dissipate intra-articular impact forces through reversible bond breakage and reformation. This physical buffering effect directly attenuates the aberrant mechanosensing of chondrocytes, preventing the force-induced activation of apoptotic pathways before biochemical drugs even take effect [28].

Furthermore, emerging evidence from mechanobiology underscores that the biomechanical function of lubrication is intrinsically linked to the biochemical resolution of inflammation, representing a synergistic mechanism of micro-nano platforms [119,120]. The reduction of interfacial friction achieved by these systems does not merely alleviate physical wear but actively interrupts pro-inflammatory signaling cascades. Specifically, excessive mechanical stress and shear forces under low-lubrication conditions activate mechanosensitive ion channels (e.g., TRPV4) in chondrocytes and synoviocytes, which in turn propagate the NF-κB pathway—a master regulator of inflammatory responses that upregulates cytokines (e.g., IL-1β, TNF-α) and matrix-degrading enzymes (e.g., MMP-13) [[121], [122], [123]]. By re-establishing a low-friction interface, micro-nano composite structures mitigate this mechanical activation at its source. Beyond attenuating mechanotransduction, recent findings underscore that the lubrication layer itself serves as a critical “biophysical shield.” Xiao et al. proposed a “Lubrication Barrier” strategy using collagen-targeting liposome-functionalized microspheres (CL@Lipo-w@AHAMs). They demonstrated that the dense hydration shell formed on the cartilage surface does more than reduce the friction coefficient to 0.036; it physically blocks the convective transport of inflammatory mediators from the synovial fluid into the deep matrix. This mechanism effectively severs the “inflammatory communication” between the hostile joint environment and the regenerating tissue, providing a quiescent niche for repair [124].

Beyond simple wear protection, the therapeutic efficacy of advanced lubrication is increasingly recognized as a mechanobiological intervention. Micro-nano platforms facilitate the biochemical resolution of inflammation by intercepting physical stimuli at the interface. Specifically, the reduction in friction coefficient directly modulates mechanosensitive pathways, such as the TRPV4-mediated calcium signaling and the subsequent NF-κB inflammatory cascade [123,125]. By dampening these mechanical-to-biochemical transduction events, micro-nano lubricants effectively downregulate the expression of MMP-13 and ADAMTS-5, thereby halting the deleterious feedback loop where mechanical shear exacerbates biochemical catabolism. This integrated 'physical-biochemical' synergy transitions the role of micro-nano structures from passive lubricants to active immunomodulatory regulators.

Consequently, this “mechano-immunomodulatory” mechanism demonstrates that the biomechanical support provided by micro-nano systems is not ancillary but fundamental. By simultaneously reducing physical wear (Lubrication), dissipating impact energy (Shock Absorption), and blocking inflammatory mediators (Barrier), these platforms reprogram the OA joint towards a restorative state [126,127].

### 3.3 Immunomodulation: orchestrating the pro-inflammatory to pro-regenerative switch

#### 3.3.1 Macrophage reprogramming

These platforms reverse chronic OA inflammation through immune-cell reprogramming. Magnetically guided microspheres (containing SPIONs) or IL-10-loaded Janus particles enable spatial guidance and phenotypic modulation of synovial macrophages [56,79]. For example, itaconate-loaded MOF-hydrogel systems activate the Nrf2/KEAP1 pathway, increasing the proportion of reparative M2 macrophages from 32.1% to 78.4% and significantly suppressing TNF-α and IL-6 expression [54,84].

Beyond chemical signaling, recent advances highlight that the physical properties of biomaterials—including stiffness, nanotopography, and porosity—serve as critical immunomodulatory cues that can independently direct macrophage polarization, a concept now recognized as “immuno-materials” engineering [128]. For instance, material stiffness (Young's modulus) directly influences macrophage mechanosensing: substrates with intermediate stiffness (∼10–20 kPa) tend to promote M2 polarization through mechanosensitive ion channel activation, while stiffer materials may favor pro-inflammatory M1 phenotypes [128]. Similarly, hierarchical surface texture—such as nanoscale roughness or specific pore architectures—can modulate macrophage morphology and cytokine secretion profiles through integrin-mediated signaling [129,130]. Multiple studies have demonstrated that larger pore structures tend to induce macrophage polarization toward an anti-inflammatory, pro-repair M2 phenotype [131,132]. For instance, implant materials with pore sizes exceeding 20 μm have been associated with a shift toward the M2 phenotype. In one study, microspheres with a diameter of approximately 146 μm predominantly accommodated M2 macrophages, whereas smaller microspheres (around 48 μm) maintained a higher proportion of M1 macrophages. These physical cues operate in concert with, or sometimes independently of, the biochemical payload, suggesting that the material's physical architecture itself is an active participant in immune reprogramming. Integrating this perspective into nanomedicine design for OA treatment could enable more precise control over the inflammatory microenvironment by simultaneously leveraging both chemical and physical immunomodulatory strategies. Beyond directly targeting terminal effector cells like macrophages, recent paradigm shifts have focused on manipulating upstream immune regulators to achieve “Plasticity Engineering.” A prime example is the modulation of Myeloid-Derived Suppressor Cells (MDSCs), which possess a paradoxical dual role in OA pathology: driving Th17-mediated inflammation while simultaneously possessing the potential to induce M2 macrophage polarization. To harness this duality, Guo et al. developed a bio-responsive HAMA-microsphere system integrated with functionalized mesoporous silica nanoparticles (MSNs). By co-delivering anti-IL-1β and TGF-β1, this “micro-nano logic” platform precisely decoupled the bifunctional roles of MDSCs: it selectively blocked the MDSC-driven differentiation of pathogenic Th17 cells while amplifying their intrinsic capacity to drive M2 macrophage polarization. This strategy exemplifies a sophisticated “Upstream-to-Downstream” immune decoding approach, where micro-nano composites act not merely as drug carriers, but as intracellular fate-switchers that redirect the pathological cellular hierarchy toward a regenerative trajectory [133].

#### 3.3.2 Metabolic homeostasis and redox regulation

**Fig. 7:**
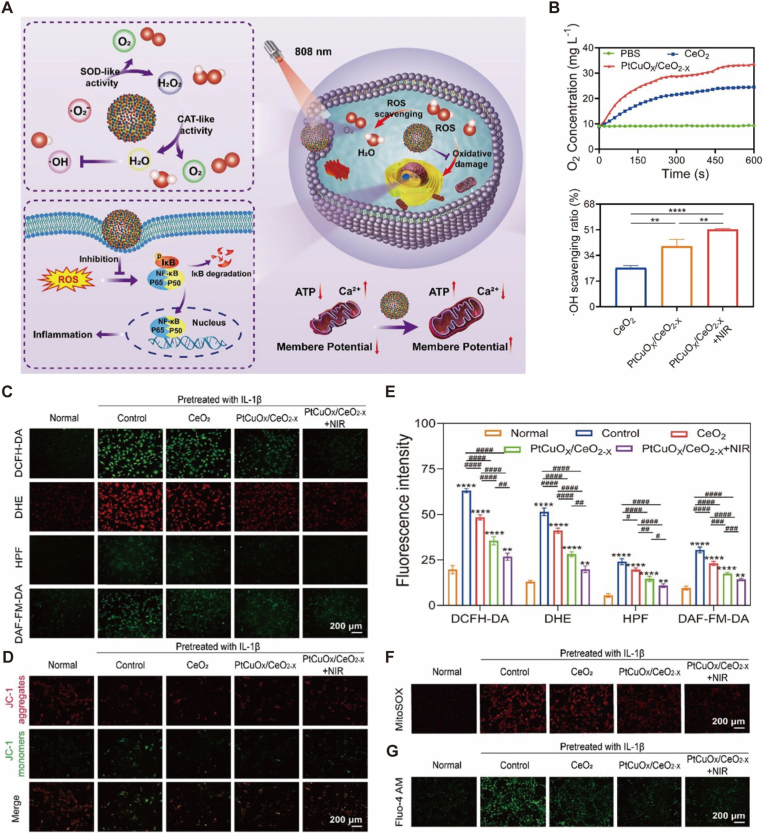
Micro/nanostructured nanozymes regulating oxidative metabolism and oxygen supply for osteoarthritis therapy. (A) Schematic illustration of the therapeutic mechanism of oxygen vacancy-engineered PtCuOX/CeO2-X nanospheres. The micro/nanostructure features abundant active sites that mimic SOD and CAT activities, converting harmful intracellular ROS (e.g., O2-,H2O2) into H2O and O2, thereby replenishing the oxygen supply and remodeling the inflammatory microenvironment under NIR irradiation. (B) Quantitative analysis showing the oxygen generation capacity (top) and hydroxyl radical (·OH) scavenging efficiency (bottom) of the nanospheres. Note that the defect-engineered structure significantly promotes O2 production via CAT-like activity. (C, E) Fluorescence images and corresponding quantitative analysis of intracellular ROS levels (DCFH-DA, DHE, HPF, and DAF-FM-DA) in chondrocytes, demonstrating the broad-spectrum antioxidative capability of the nanostructure. (D, F, G) Evaluation of mitochondrial oxidative metabolism recovery. (D) JC-1 staining indicating the restoration of mitochondrial membrane potential (red fluorescence aggregates). (F) Mitigation of mitochondrial superoxide (MitoSOX). (G) Regulation of intracellular Ca2+ homeostasis (Fluo-4 AM). The engineered micro/nanostructures not only scavenge ROS to alleviate oxidative stress but also actively generate oxygen to rescue mitochondrial metabolic function, inhibiting chondrocyte apoptosis. Reproduced under terms of the CC-BY-NC-ND license[135]. Copyright 2024, The Authors, published by Springer Nature.

Emerging therapeutic paradigms have shifted focus from symptom suppression to fundamental “Metabolic Reprogramming.” As comprehensively defined in a recent review by Cheng et al., next-generation “Metabolism-Regulating Microspheres” are designed to correct the triad of metabolic abnormalities—glycolytic dysregulation, mitochondrial dysfunction, and oxidative stress—that drive OA pathogenesis. Within this framework, specific subcellular organelles have become precise targets for intervention [134]. At the cellular level, the restoration of metabolic homeostasis is driven by the synergistic regulation of signaling molecules and oxidative status. MSC-EVs deliver specific miRNAs (e.g., miR-92a-3p) to improve mitochondrial function, effectively increasing ATP production by 230% [17]. Simultaneously, micro/nanostructured nanozymes, such as the oxygen vacancy-engineered PtCuOX/CeO2-X nanospheres, provide a sophisticated dual-action mechanism (Fig. 7A). These engineered units mimic natural antioxidant enzymes (SOD and CAT) through valence switching, which not only scavenges hydroxyl radicals and other ROS with high efficiency (Fig. 7B; Fig. 7E) but also actively generates oxygen from harmful H2O2 (Fig. 7B) [82]. Expanding this catalytic repertoire to bioactive hybrids, Feng et al. engineered Chondroitin Sulfate-based microspheres loaded with Ganoderma Lucidum Polysaccharide-functionalized MnO2 nanozymes. This system couples manganese-driven oxygen generation with polysaccharide-mediated immunomodulation, effectively alleviating the hypoxic blockade that restricts M2 macrophage polarization [72].

This active oxygen replenishment is critical for reversing the hypoxic stress within the OA microenvironment, as evidenced by the restoration of mitochondrial membrane potential (Fig. 7D) and the regulation of intracellular Ca2+ homeostasis (Fig. 7G). By mitigating mitochondrial superoxide levels (Fig. 7F) and rescuing oxidative metabolism, these systems effectively inhibit chondrocyte apoptosis and promote matrix synthesis. Distinct from artificial enzyme mimics, strategies leveraging trace element supplementation aim to reinvigorate endogenous antioxidant defenses. Liu et al. identified Selenium (Se) deficiency as a critical bottleneck in mitochondrial redox homeostasis. They developed a “Cascade Targeting” strategy, utilizing hydrogel microspheres to deliver hyaluronic acid-modified Selenium Nanoparticles (SeNPs) deep into chondrocyte mitochondria. This approach directly supplements the essential co-factor required for selenoprotein synthesis (e.g., GPX1, TrxR2), thereby rescuing mitochondrial oxidative phosphorylation and correcting the redox imbalance at its nutritional root [[136], [137], [138]]. Such multiscale orchestration aligns with the paradigm shift toward “subcellular therapy,” where micro-scale carriers ensure joint retention while nano-units specifically rescue mitochondrial bioenergetics [139]. Deepening the intervention into redox homeostasis, recent research has expanded focus from apoptosis to blocking Ferroptosis—an iron-dependent, lipid peroxidation-driven form of regulated cell death. Wang et al. identified that the downregulation of the GPX4/SLC7A11 axis is a critical driver of chondrocyte loss. To reverse this, they engineered a “reverse-adaptation” strategy: MSCs were preconditioned under hypoxia to secrete exosomes enriched with anti-ferroptotic cargo. When encapsulated in hydrogel microspheres to ensure sustained bioavailability, these “super-exosomes” significantly upregulated GPX4 expression, effectively arresting the ferroptotic cascade that conventional antioxidants often fail to halt [140]. Parallel to mitochondrial rescue, alleviating Endoplasmic Reticulum (ER) stress represents another frontier in subcellular precision therapy. Excessive mechanical load triggers the accumulation of unfolded proteins, activating the Unfolded Protein Response (UPR) and pro-apoptotic factors like CHOP. To counteract this, TUDCA-loaded cationic liposomes, when encapsulated within stress-relaxing microspheres, act as chemical chaperones. By targeting the Wyrgrl peptide to collagen II, these nanounits facilitate precise intracellular delivery, significantly downregulating GRP78 and CHOP expression to restore proteostatic homeostasis [28]. Extending beyond organelle stress, the restoration of “temporal homeostasis” (Circadian Rhythm) represents a novel dimension, yet it demands sustained intervention to effectively entrain the dysregulated molecular clock. Chondrocytes possess an intrinsic clock (e.g., BMAL1/CLOCK) that governs metabolism, but short-lived therapeutics often fail to reset stable rhythms. Addressing this, Yan et al. pioneered a strategy using plant-derived nanovesicles (WELNs). Crucially, to prevent rapid articular clearance, these nanounits were encapsulated within MMP-responsive microgels. This hierarchical design transformed the transient nanovesicles into a sustained “temporal modulator,” effectively resynchronizing the circadian rhythm to suppress inflammation and promote matrix anabolism [141]. Clinical translational studies indicate that such advanced exosome-microsphere or nanozyme systems (e.g., microenvironment remodeling platforms) can improve WOMAC scores by 41% compared to conventional hyaluronic acid treatments [77,139], demonstrating profound DMOAD potential through the comprehensive remodeling of the joint's redox and metabolic landscape.

## 4 Micro-nano platforms: multifaceted roles in cartilage regeneration

### 4.1 Biomimetic scaffolds for niche recapitulation

The therapeutic efficacy of micro-nano composite structures lies in their ability to recapitulate the native articular cartilage extracellular matrix across multiple scales, thereby providing an integrated biomimetic platform for structural and functional restoration. In terms of structural biomimicry, native cartilage consists of a hierarchical meshwork of nanoscale collagen fibers and microscale porous proteoglycan matrices. Conventional single-scale scaffolds often fail to balance the requirements for cell infiltration and phenotype maintenance. To address this, electrospun nanofibers (e.g., PLGA/PCL, 100–500 nm in diameter) are employed to mimic the topological cues of native collagen, facilitating chondrocyte adhesion via integrin-mediated physical anchoring. Concurrently, the integration of microscale pores (50–200 μm) within the scaffold framework provides essential conduits for nutrient diffusion and metabolic exchange, effectively replicating the heterogeneous porosity of native tissue [18,53,80].

Beyond structural guidance, the biomechanical adaptation of these platforms is crucial for enduring the complex intra-articular stress environment. While early synthetic scaffolds often exhibited a mechanical mismatch with native cartilage due to their excessive rigidity and lack of energy-dissipating capacity, next-generation “sponge-hydrogel” composites (e.g., SponGel MS) offer a solution. In these systems, a microscale framework provides the requisite load-bearing stiffness, while the embedded nanoscale hydrogel mimic the viscoelastic and energy-absorbent properties of cartilage by fine-tuning crosslinking densities [34,39]. Although the incorporation of nanoclay and other reinforcing units has aligned the modulus (0.5–2 MPa) of these composites with the biological window of cartilage, their long-term fatigue resistance and interfacial stability under chronic cyclic loading remain critical hurdles for clinical translation.

Building upon physical support, the functional integration strategies of micro-nano systems are shifting the paradigm of cartilage repair from passive replacement to active induction. Interfacial modifications, such as nanocoatings of hyaluronic acid or chitosan, can activate the CD44 signaling pathway through ligand-receptor interactions, thereby enhancing chondrocyte proliferative activity [39,142]. Furthermore, microsphere-based delivery systems encapsulating growth factors (e.g., TGF-β3, KGN) or genetic payloads (e.g., SOX9) offer precise temporal control over signaling cascades, overcoming the limitations of short half-lives and rapid clearance associated with free therapeutic agents. A primary advantage of such hierarchical logic is the ability to orchestrate chondrocyte extracellular matrix synthesis (e.g., COL2 and ACAN) by activating key transcriptional regulators like the SOX9 pathway (Fig. 4C). A notable example is the WYRGRL-DOTAP-Lipo@KGN@GM system developed by Li et al., which harmonizes targeted nanoliposomes with mechanical-supporting microgels to achieve a 2.1-fold upregulation in COL2A1 expression [54,57]. While these multiscale strategies demonstrate superior chondrogenic potential in experimental models, a critical appraisal is necessary regarding the long-term stability of these bio-signals within the harsh OA inflammatory milieu and the potential chronic biosafety risks posed by exogenous nanoparticles, necessitating further validation in large animal models.

### 4.2 Cell delivery platforms: orchestrating spatiotemporal cell behavior

Micro-nano composite carriers, characterized by their hierarchical “micro-scaffold/nano-cargo” architecture, achieve a sophisticated synergy of cell protection, controlled drug release, and spatial adaptation, driving a paradigm shift in OA therapy from passive filling to active modulation. In terms of delivery logic, these systems leverage the complementary advantages of multiscale physicochemical properties. Microscale frameworks (e.g., PLGA or porous HAMA/GelMA microspheres) provide essential mechanical support and 3D anchorage zones for seed cells, while functioning as physical barriers that block approximately 70% of pro-inflammatory M1 macrophage infiltration. This creates localized “immune-privileged niches” within the hostile intra-articular environment [43,47]. Simultaneously, integrated nanounits ameliorate oxidative stress through ROS scavenging or activate chondrogenic pathways (e.g., Wnt/β-catenin) to direct cellular differentiation [24,44]. To address the clinical challenge of rapid therapeutic clearance, magnetic micro-nano systems assisted by external fields have successfully extended cell residence time to 14 days, significantly enhancing targeted enrichment at lesion sites [55,56].

Regarding the precision induction of cellular behavior, micro-nano carriers demonstrate exceptional spatiotemporal programming capabilities. Unlike conventional single-stage carriers prone to burst release, hybrid NiMs systems utilize hierarchical encapsulation to retard drug diffusion, ensuring the sustained, steady-state release of growth factors such as BMP-2 [59]. This regulatory capacity extends to the genetic and nucleic acid levels. While earlier strategies utilized plasmid-loaded nanoparticles to upregulate regenerative markers (e.g., SOX9) [58], recent advances have focused on post-transcriptional silencing. For instance, Yang et al. engineered a 'dual-lock' system by encapsulating chondrocyte-targeting exosomes within GelMA microspheres. This hierarchical platform not only achieved sustained intra-articular retention but also precisely delivered miR-148a into chondrocytes, effectively reversing the abnormal downregulation of this miRNA and suppressing cartilage matrix loss [143]. The transition from passive filling to active modulation is prominently evidenced by MN-mediated biochemical signaling. A quintessential example is the use of PDA@Exo-loaded MNs, which bypass traditional delivery barriers to establish a sustained signaling hub. These systems exert active modulation by activating the PI3K-Akt-mTOR pathway in chondrocytes [108,144], effectively shifting cellular metabolism from a catabolic inflammatory state toward regenerative anabolism. This precise 'fate modulation' ensures that the delivered bioactives or local cells are directionally programmed for chondrogenic differentiation rather than terminal hypertrophy [145]. The emergence of “logic-gated” systems further refines this intelligence. By specifically recognizing the acidic milieu (pH < 6.5) or overexpressed enzymes (e.g., MMP13) in OA joints, these systems achieve on-demand triggering of therapeutics, thereby remodeling the localized microenvironment at the molecular level [47,54,57].

Furthermore, the sophisticated design of micro-nano architectures profoundly influences the spatial order of tissue regeneration. Gradient pore designs facilitate high-efficiency MSC seeding while optimizing nutrient diffusion paths, whereas advanced Janus architectures utilize asymmetrical interfaces to guide directional cell alignment, strengthening the biomechanical integration of the osteochondral interface [18,79]. Nevertheless, from a clinical translation perspective, most current studies focus on modulating single signaling pathways. Under the multifaceted pathological conditions of real-world OA, critical issues such as physical interference between different nanounits' release kinetics, the risk of microsphere fragmentation under high-frequency joint motion, and the chronic irritation of synovium by long-term degradation products remain formidable hurdles. While the transition from “space-filling” to “fate modulation” is promising, future research must prioritize maintaining structural consistency and dynamic mechanical adaptation during large-scale manufacturing.

### 4.3 Endogenous repair: from spatiotemporal recruitment to in situ regeneration

Micro-nano composite structures leverage the precise spatiotemporal release of biochemical signals to mobilize the host's intrinsic reparative potential, effectively circumventing the immunogenic risks and ethical constraints associated with exogenous cell transplantation. In the context of endogenous stem cell recruitment and homing, these carriers act as pivotal “chemotactic signal sources.” For instance, composite microspheres loaded with SDF-1 utilize nanoscale reservoirs to achieve the sustained release of chemokines over 30 days, establishing a stable concentration gradient that enhances the migration efficiency of synovial mesenchymal stem cells (SMSCs) [47,103]. Simultaneously, environmentally responsive nanocarriers, such as MnO_2_@EGCG, exert multifaceted effects within the lesion: they alleviate oxidative stress by scavenging superoxide anions while sequentially delivering chondrogenic factors like TGF-β to provide essential lineage-specification cues for recruited endogenous cells [55,105].

**Fig. 8:**
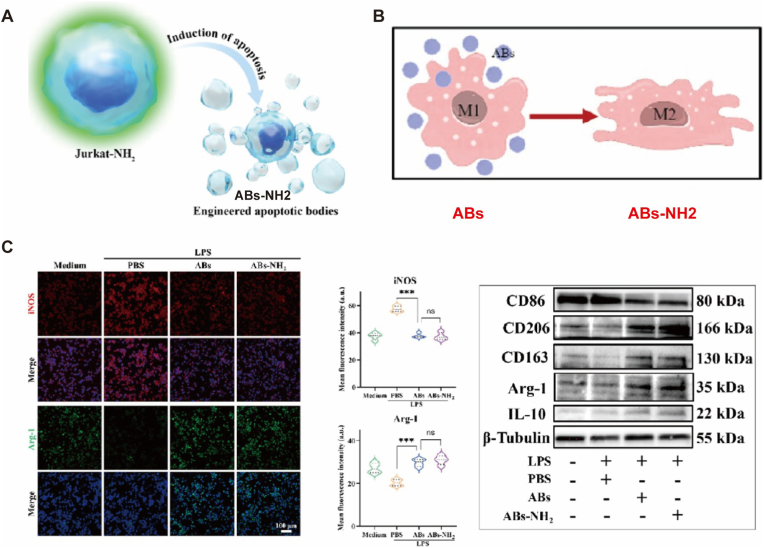
Lymphocyte-derived engineered apoptotic bodies as bioactive cellular messengers for immunomodulatory signal delivery. (A) Schematic illustration of the engineering and generation process. Jurkat T cells are surface-modified with amino groups (-NH2) and induced to undergo apoptosis, yielding engineered apoptotic bodies (ABs-NH2) that serve as micro/nano-scale cellular signal carriers. (B) Mechanism of signal transduction. The apoptotic bodies are engulfed by pro-inflammatory M1 macrophages, delivering intracellular signals that trigger a phenotypic switch to the anti-inflammatory M2 state, thereby resolving osteoarthritis-associated inflammation. (C) In vitro validation of signal delivery efficacy. Immunofluorescence staining (left) and quantitative analysis (middle) demonstrate that ABs- NH2 treatment significantly downregulates the M1 marker iNOS (red) while upregulating the M2 marker Arg-1 (green). Western blotting (right) further confirms the successful delivery of immunomodulatory signals, indicated by the increased expression of regenerative proteins (CD206, CD163, Arg-1, IL-10) and suppression of inflammatory markers (CD86). These results confirm that engineered apoptotic bodies function as effective biological vehicles to deliver cues that reprogram the immune microenvironment. Reproduced with permission[101]. Copyright 2024 American Chemical Society.

The profound remodeling of the microenvironment serves as the tipping point for shifting endogenous repair from “inflammatory degradation” toward “tissue regeneration.” Immunomodulatory micro-nano scaffolds, such as the KM13E system delivering IL-10+ EVs or engineered cellular messengers like amino-functionalized apoptotic bodies (ABs-NH2) (Fig. 8A), utilize bioactive molecules to drive macrophage polarization from a pro-inflammatory M1 phenotype to a pro-regenerative M2 phenotype (Fig. 8B). Experimental validation shows that such engineered carriers significantly downregulate M1 markers (iNOS) while upregulating M2 markers (Arg-1) (Fig. 8C, left), increasing the expression of regenerative proteins such as CD206 and IL-10 (Fig. 8C, right) to reconstruct an anti-inflammatory niche in situ [54,101]. Furthermore, micro-nano systems doped with metal ions (e.g., Mg^2+^) activate the HIF-1α pathway in endogenous cells, enhancing their anabolic metabolism and chondrogenic potential under hypoxic conditions [81]. Experimental evidence indicates that this in situ regeneration strategy offers superior targeting precision compared to conventional cell therapies, with a single injection increasing cell density in defect areas and significantly reducing OARSI scores [44,58,77,146].

In the context of stimulating endogenous repair, MN platforms function as intelligent delivery depots for chemotactic agents. By creating micro-channels and releasing recruitment factors (e.g., -linolenic acid or growth factors) in a controlled manner [38], MNs can effectively mobilize endogenous mesenchymal stem cells (MSCs) to the lesion site. This approach facilitates in situ remodeling without the need for exogenous cell transplantation, leveraging the synergistic effects of minimally invasive physical stimulation and localized biochemical recruitment to enhance the joint's innate regenerative capacity.

**Fig. 9:**
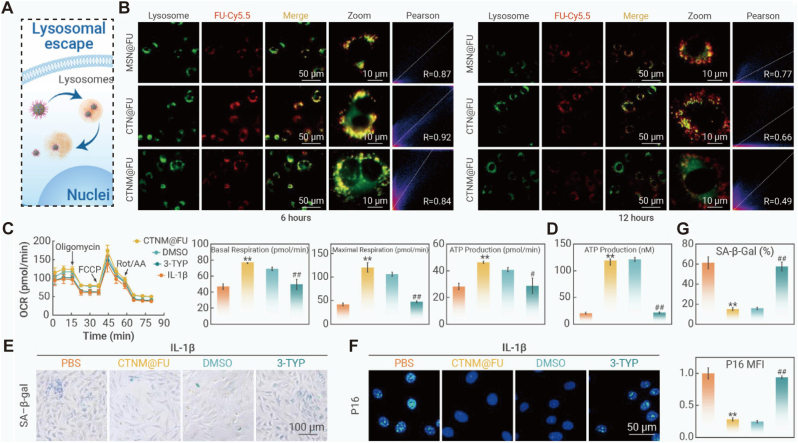
Revitalizing endogenous chondrocytes via organelle-targeted micro/nanotherapeutics. (A) Schematic illustration of the “Trojan horse” strategy. The cationic nanoparticle-hydrogel composite (CTNM@FU) facilitates lysosomal escape to deliver fucoidan (FU) intracellularly, targeting mitochondrial homeostasis. (B) Confocal laser scanning microscopy images tracking the intracellular trafficking of Cy5.5-labeled FU (red) and lysosomes (green). The decreased Pearson’s correlation coefficient in the CTNM@FU group (bottom row) indicates the successful escape of the payload from lysosomes (“proton sponge effect”), preventing enzymatic degradation. (C-D) Metabolic analysis showing the oxygen consumption rate (OCR) and ATP production in chondrocytes under inflammatory conditions (IL-1β). Note that the delivery system reverses mitochondrial dysfunction, restoring energy metabolism to physiological levels. (E-G) Evaluation of cellular senescence via SA-β-Gal staining and P16 immunofluorescence. The restoration of mitochondrial energy production effectively impedes chondrocyte senescence (blue staining), validating the potential of this cell-free strategy to rejuvenate host tissues by manipulating organelle function. Reproduced under terms of the CC-BY-NC-ND license[23]. Copyright 2025, The Authors, published by Elsevier.

In terms of clinical application and metabolic homeostasis, the “Trojan Horse” microsphere (CTNM@FU) developed by Lu et al. exemplifies the advantages of endogenous activation by facilitating lysosomal escape to target mitochondrial homeostasis (Fig. 9A). Confocal tracking of the intracellular trafficking of Cy5.5-labeled payloads reveals a significantly decreased Pearson's correlation coefficient over 12 h, validating the successful escape of fucoidan from lysosomes via the “proton sponge effect” to prevent enzymatic degradation (Fig. 9B). By triggering the pH-responsive release of fucoidan, this system activates the SIRT3 signaling pathway in resident chondrocytes, reversing mitochondrial dysfunction by restoring the oxygen consumption rate (OCR) and boosting ATP production to physiological levels (Fig. 9C and D) [44,147]. Notably, this restoration of mitochondrial energy production effectively impedes chondrocyte senescence, as evidenced by the marked reduction in SA-β-gal staining and P16 immunofluorescence intensity (Fig. 9E–G). Although cell-free therapy demonstrates immense translational potential, a critical appraisal suggests that the efficacy of endogenous repair is heavily contingent upon the “residual progenitor cell pool” within the patient's joint—a factor that may be severely compromised in elderly patients with advanced OA. Moreover, ensuring that recruited cells avoid “fibrotic degeneration” in a hostile inflammatory milieu and maintaining the bioactivity of chemotactic signals over extended recruitment cycles remain significant technical hurdles for future investigation.

### 4.4 Theranostic applications: synchronizing diagnosis and treatment

The integration of diagnostic and therapeutic functionalities within micro-nano composite structures offers a transformative approach to managing osteoarthritis and rheumatoid arthritis, enabling a seamless transition from real-time monitoring to localized, high-fidelity intervention. By leveraging biomarker-responsive sensing, multi-modal imaging guidance, and integrated microneedle platforms, these systems effectively bridge the gap between early detection and precision therapy.

#### 4.4.1 Biomarker-triggered sensing and precise release

The efficacy of micro-nano theranostics resides in their capacity to function as “intelligent reporters” that respond to specific pathological hallmarks of the arthritic microenvironment, such as overexpressed ROS, matrix metalloproteinases (notably MMP-13), and acidic pH fluctuations. For instance,

ROS-responsive nanopatterns, such as thioketal-linked TKCP@DEX nanoparticles, exhibit a “turn-on” fluorescence signal upon exposure to oxidative stress in OA joints, facilitating on-demand dexamethasone release and accurate disease grading [148]. Similarly, dual MMP-13/pH-responsive systems, exemplified by cartilage-targeting CMFn@HCQ ferritin cages, enable synchronized hydroxychloroquine delivery and visualization specifically within the acidic, enzyme-rich synovial niche [149]. Furthermore, enzyme-triggered strategies using MMP-13-sensitive peptide-modified KM13E@PGE hydrogel microspheres allow for the staged release of IL-10+ and SOX9+ extracellular vesicles, sequentially addressing inflammation and promoting chondrogenic remodeling [54].

#### 4.4.2 Multi-modal imaging guidance

Real-time visualization of therapeutic distribution and tissue response is essential for optimizing treatment regimens. Micro-nano composites have integrated diverse contrast agents to enable sophisticated guidance through varied imaging modalities. In the treatment of RA, quadrate ruthenium nanoparticles (QRuNPs) encapsulated within QRu-PLGA-RES-DS nanocomposites utilize near-infrared-II (NIR-II) photoacoustic imaging (PAI) to guide therapy while simultaneously inducing M2 macrophage polarization [150,151]. For earlier-stage OA diagnosis, manganese oxide-based nanoparticles (WY-CMC-MnOx NPs) provide enhanced T1-weighted magnetic resonance imaging (MRI) contrast alongside cartilage repair functionalities, significantly improving the sensitivity of joint assessments [152]. Additionally, by constructing a 'Bubble-in-Microgel' hierarchical architecture, Han et al. [47] developed ultrasonic contrast microspheres loaded with IL-10 nanobubbles. This system not only enhances acoustic echogenicity for precise synovitis visualization but also enables ultrasound-triggered, macrophage-targeted drug release, realizing real-time monitoring of inflammation resolution(Fig. 3A–C).

#### 4.4.3 Integrated diagnostic-therapeutic microneedles

The evolution of microneedle (MN) systems has established a minimally invasive interface that combines diagnostics with drug delivery, significantly enhancing patient compliance compared to repeated intra-articular injections. Dissolvable MNs, such as hyaluronan-based arrays loaded with celecoxib nanocrystals (CXB-NC@DMNs), provide efficient transdermal delivery that maximizes bioavailability and provides potent analgesic effects [36]. Advanced photothermal-responsive MNs (e.g., PDA@Exo MNs) have demonstrated the ability to inhibit cartilage degradation via the PI3K-Akt-mTOR signaling pathway while utilizing NIR imaging to monitor localized drug distribution [108]. Moreover, dual-functional MNs, such as the HA-DCF@PDMPC bilayer system, reduce insertion trauma through lubricating coatings and achieve multi-stage diclofenac release via ester bond hydrolysis [110].

In summary, micro-nano composite structures have successfully synchronized biomarker-activated sensing, imaging navigation, and microneedle-based intervention to realize precision medicine for arthritis. Future research must continue to prioritize the optimization of biocompatibility and the standardization of large-scale fabrication processes to accelerate the clinical translation of these intelligent theranostic platforms [30,153].

## 5 Current challenges and future perspectives

Micro-nano composite structures have demonstrated immense potential in the field of precision medicine for osteoarthritis, evolving from simple drug delivery vehicles into multifunctional integrated diagnostic and therapeutic platforms. However, bridging the gap between bench-top research and large-scale clinical application necessitates overcoming multilayered bottlenecks in standardized manufacturing, dynamic bio-sensing, and interdisciplinary integration.

### 5.1 Rational design: balancing performance gains with engineering hurdles

The evolution of micro-nano composite structures in osteoarthritis therapy is driven by the need to resolve the “retention-penetration” paradox—a fundamental limitation of mono-scale systems. Mono-scale nanocarriers (<200 nm), while effective for cellular internalization and matrix penetration [149,154], are susceptible to rapid lymphatic clearance, with synovial half-lives often falling below 24 h [55,59,155]. Conversely, mono-scale microparticles (>10 μm) provide extended joint residency [10,59] but are physically excluded from the dense cartilage extracellular matrix, confining their action to the superficial synovial layer [154,156]. Micro-nano hierarchical systems bypass this through a staged-delivery strategy: micro-scale matrices (e.g., chitosan or magnetic PLGA microspheres) resist clearance to maintain an extended residence [55,56,59], while encapsulated nanounits serve as “penetration units” that infiltrate the deep cartilage matrix upon pathological triggers such as pH, MMP-13, or ROS [105,148,149,157]. Quantitative assessments confirm that such hierarchical designs significantly outperform mono-scale systems in improving structural repair indices (e.g., OARSI scores) and maintaining effective drug concentrations [36,37,47,54,75,110,158].

Despite these performance gains, the “complexity tax” associated with multi-layered systems necessitates a rigorous evaluation of their clinical utility. In acute traumatic scenarios, rapid suppression of inflammation via simple nanocarriers or small molecules may be prioritized over long-term tissue remodeling. For instance, while composite systems like the HA-DCF@PDMPC microneedle patch maintain drug levels for 72 h compared to 24 h for mono-scale systems [37], this advantage may be neutralized in short-term anti-inflammatory interventions where self-assembled drug microparticles (e.g., methylprednisolone sucinate) already provide sufficient efficacy [8]. Furthermore, the engineering hurdles of scaling up hierarchical designs cannot be overlooked. The multi-step fabrication of composites, such as loading nanoparticles into PLGA microspheres, increases the risk of batch-to-batch variability compared to standardized mono-scale carriers [56,60]. The cumulative metabolic burden is also a concern; the acidic degradation of polyester matrices like PLGA may exacerbate synovial inflammation [55,159], whereas natural polymers like hyaluronic acid or chitosan offer a milder degradation profile [47,160].

Closely intertwined with these metabolic concerns is the frequently overlooked challenge of precisely synchronizing the degradation kinetics of the micrometer-scale matrix with the release profile of the encapsulated nano-cargo. An ideal scenario requires the scaffold's structural integrity to persist just long enough to facilitate neo-tissue formation while ensuring a sustained, non-burst release of therapeutic nanoparticles. Mismatched timelines can lead to significant drawbacks: if the micro-matrix degrades too rapidly, it can result in a premature and massive “burst release” of nanounits, leading to off-target effects and a short therapeutic window [42,55]. Conversely, a matrix that degrades too slowly may physically hinder tissue integration, potentially leading to fibrous encapsulation and suboptimal repair outcomes [53]. Achieving such synchronized behavior typically requires moving beyond simple physical blending towards intelligent chemical linkages, such as covalent grafting or enzymatic-responsive coupling, to ensure the degradation of the macro-scaffold and the activation of the nano-therapeutics are cohesively programmed [15,94].

Beyond manufacturing and metabolic considerations, future material design must explicitly resolve the “injectability-performance paradox.” To ensure clinical viability, balancing structural complexity with shear-thinning properties is essential for enabling minimally invasive delivery through fine-gauge needles (e.g., 25-27 gauge). Promising strategies include optimizing carrier morphology via microfluidics to produce monodisperse, smooth-surfaced microspheres that minimize inter-particle friction, and surface functionalization with hydrophilic polymers (e.g., PEG) to reduce viscosity. Furthermore, formulating micro-nano carriers within shear-thinning hydrogels (e.g., hyaluronic acid derivatives or nanoclay-reinforced gels) allows these vehicles to facilitate smooth flow during injection while ensuring rapid structural recovery post-injection to maintain intra-articular retention [161,162].

Consequently, this review advocates for the “Rational Complexity” design principle: micro-nano integration should be prioritized only when biological barriers cannot be breached by mono-scale systems. Specifically, hierarchical designs are essential when deep cartilage penetration requires avoiding rapid synovial clearance (half-life <24 h) [149,155] or when spatiotemporal synergy is required to target both the synovium at the macro-scale and chondrocytes at the nano-scale [47,57,156]. By adhering to the principle of necessity, researchers can balance therapeutic gains with complexity costs, utilizing advanced techniques like microfluidics [53,66] and biocompatible natural polysaccharides [160] to minimize potential risks and accelerate the clinical translation of these intelligent platforms [163,164].

### 5.2 Evolution toward dynamic responsiveness and “closed-loop” control

Most current micro-nano systems rely on preset release kinetics and lack the capacity for dynamic adaptation to the spatiotemporal fluctuations of joint inflammation. Future intelligent designs must focus on logic-gated feedback systems. For instance, nanocarriers utilizing dual ROS/pH-sensitive bonds (e.g., arylboronate esters) can achieve triggered release specifically during inflammatory peaks, thereby avoiding off-target drug delivery during quiescent periods [57,106]. A more visionary approach involves the interdisciplinary integration of closed-loop biosensing systems. By incorporating flexible electronic sensors (e.g., graphene arrays) or bio-responsive components within MN patches to monitor pro-inflammatory cytokine fluctuations (such as IL-1β) in real-time, the system can dynamically adjust the therapeutic release rate. Owing to their minimally invasive penetration of the tissue surface, MNs provide a superior interface for integrating wearable sensors with localized nano-carriers, enabling a “monitoring-feedback-execution” loop [36,38]. Such integration will shift OA intervention from passive defense to autonomous regulation, marking a transition toward personalized and intelligent bio-micro-nano systems [57].

### 5.3 Interdisciplinary synergy and paradigm shifts

Artificial Intelligence (AI) and Bioinformatics-driven discovery are reshaping the material design paradigm. AI-driven material prediction leverages machine learning models (e.g., Generative Adversarial Networks, GANs) to establish non-linear mappings between physical parameters (e.g., pore size, modulus) and stem cell fates, significantly accelerating the screening of biomimetic scaffolds [66]. AI models (e.g., GANs) accelerate scaffold screening [46], while causal inference methodologies like Mendelian Randomization (MR) offer a robust framework for upstream target identification. For instance, Yan et al. utilized MR macroanalysis to unveil the causal genetic link between specific gut flora and OA, precisely pinpointing therapeutic targets prior to material fabrication. This integration of genetic macroanalysis with micro-nano engineering exemplifies a “Data-to-Drug” closed-loop strategy, reducing trial-and-error redundancy. Furthermore, radiomics combined with magnetic resonance imaging facilitates the real-time tracking of SPION-labeled microspheres' metabolic patterns [61]. To address the exhaustion of the endogenous stem cell pool in elderly patients, engineered exosomes or apoptotic bodies (e.g., T-cell-derived eABs) can deliver regenerative microRNAs, bypassing the risks of cell transplantation and enabling efficient cell-free cartilage regeneration in situ [58,101].

In conclusion, through multiscale precision architecture and intelligent responsive design, micro-nano composite structures are leading the transition of OA therapy from symptomatic relief to functional regeneration. With breakthroughs in standardized bio-manufacturing and personalized adaptation, micro-nano carriers will form an integrated system characterized by precision, dynamism, and feedback. This evolution will not only rewrite the therapeutic landscape of OA but also establish a universal scientific paradigm for regenerative medicine in degenerative diseases [104].

## CRediT authorship contribution statement

**Zhijian Shi:** Methodology, Writing – original draft. **Jiayou Chen:** Methodology, Supervision, Writing – original draft. **Haochi Lun:** Writing – original draft. **Rongji Liang:** Methodology, Resources. **Jingtao Huang:** Methodology, Supervision. **Quan Lin:** Resources, Visualization. **Wei Li:** Conceptualization, Methodology, Supervision. **Zhenhan Deng:** Methodology, Project administration, Supervision. **Jianjing Lin:** Funding acquisition, Project administration, Resources.

## Declaration of competing interest

The authors declare that they have no known competing financial interests or personal relationships that could have appeared to influence the work reported in this paper.

## Data Availability

No data was used for the research described in the article.
